# Enhanced Interplay between Host–Guest and Spin-Crossover Properties through the Introduction of an N Heteroatom in 2D Hofmann Clathrates

**DOI:** 10.1021/acs.inorgchem.1c01925

**Published:** 2021-08-04

**Authors:** Manuel Meneses-Sánchez, Rubén Turo-Cortés, Carlos Bartual-Murgui, Iván da Silva, M. Carmen Muñoz, José Antonio Real

**Affiliations:** † Instituto de Ciencia Molecular and Departamento de Química Inorgánica, Universidad de Valencia, Catedrático Beltrán Martínez 2, Paterna, València E-46980, Spain; ‡ ISIS Neutron Facility, STFC Rutherford Appleton Laboratory, Chilton, Oxfordshire OX11 0QX, U.K.; § Departamento de Física Aplicada, Universitat Politècnica de València, Camino de Vera S/N, Valencia 46022, Spain

## Abstract

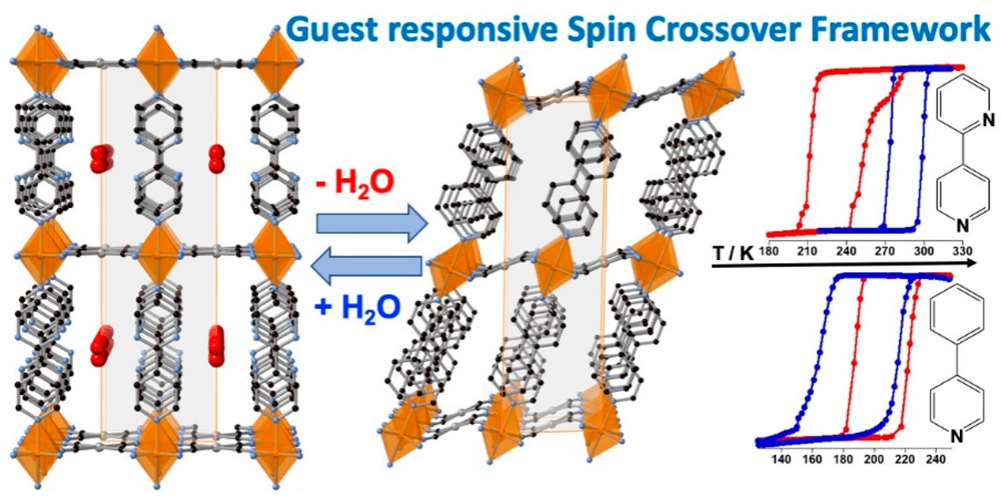

Controlled modulation of the spin-crossover (SCO) behavior through the sorption–desorption of invited molecules is an extensively exploited topic because of its potential applications in molecular sensing. For this purpose, understanding the mechanisms by which the spin-switching properties are altered by guest molecules is of paramount importance. Here, we show an experimental approach revealing a direct probe of how the interplay between SCO and host–guest chemistry is noticeably activated by chemically tuning the host structure. Thus, the axial ligand 4-phenylpyridine (4-PhPy) in the 2D Hofmann clathrates {Fe(4-PhPy)_2_[M(CN)_4_]} (**PhPyM**; M = Pt, Pd) is replaced by 2,4-bipyridine (2,4-Bipy), resulting in the isomorphous compounds {Fe(2,4-Bipy)_2_[M(CN)_4_]} (**BipyM**; M = Pt, Pd), which basically differ from the former in that they have a noncoordinated N heteroatom in the ancillary aromatic substituent, i.e., 2-pyridyl instead of phenyl. Our chemical, magnetic, calorimetric, and structural characterizations demonstrate that this subtle chemical composition change provokes outstanding modifications not only in the capability to adsorb small guests as water or methanol but also in the extent to which these guests affect the SCO characteristics.

## Abstract 2

The introduction of an N-heterocyclic atom in the aromatic interdigitated axial ligands of a 2D Hofmann-type framework provokes dramatic changes on its affinity to solvent guests. Sorption−desorption of these guests induces drastic structural changes, affecting dramatically the hysteretic spin-crossover properties of the framework.

## Introduction

The spin-crossover (SCO) behavior is essentially a molecular phenomenon exhibited by some octahedral transition-metal complexes with electronic configurations 3d^4^–3d^7^ that involves the reversible, controllable, and detectable switching between the low-spin (LS) and high-spin (HS) states.^1,2^ This switchable behavior has predominantly been studied for hexacoordinated {Fe^II^N_6_} 3d^6^ complexes because a large number of common N-donor ligands accomplish the necessary condition; i.e., the energy balance between the ligand-field strength and the interelectronic repulsion is of the order of magnitude of *k*_B_*T*.^3−5^ The HS ↔ LS switching can be triggered by a series of external stimuli (temperature, pressure, light, guest analytes, etc.), implicating remarkable variations of the optical, structural, magnetic, and electric properties. Interestingly, this external perturbation–physical signal change coupling, together with the possibility of obtaining nanometric architectures,^6^ is the basis for potential applications of SCO materials in areas of chemical sensing, switching devices, display, or information storage.^7−10^ However, from an application-perspective point of view, the SCO requires, in general, a high degree of cooperativity within the crystalline network. Cooperativity stems from long-range elastic interactions between the SCO centers in such a way that the spin-state change is efficiently transmitted within the material.^3^ Hence, cooperative thermally driven SCO materials may exhibit abrupt or even hysteretic γ_HS_ versus *T* curves (γ_HS_ = HS-state molar fraction), giving way to bistable properties. In the last 2 decades, chemists have made many efforts in order to achieve strong bistable SCO compounds. To do so, the synthesis of extended 1–3D coordination polymers (CPs), in which the SCO centers are connected by coordination/covalent bonds, has been one of the main synthetic strategies.^11,12^

Fe^II^ Hofmann-type CPs (Fe^II^-HCPs) have been one the most investigated classes of CPs within the SCO community. From the vast family of reported Fe^II^-HCPs, those frameworks presenting the general formula {Fe^II^(L)_*x*_[M^II^(CN)_4_]} (*x* = 2 or 1 for 2D or 3D systems, respectively) are constituted of bimetallic layers where the Fe^II^ ions are connected through [M^II^(CN)_4_]^2–^ anions (M = Pt, Pd, Ni).^13,14^ The cyanometallate-based layers are stacked in such a way that the organic axial ligands (L; typically substituted pyridines or triazole-based ligands) either are monodentate and interdigitated in the case of 2D networks or act as bis-monodentate bridges between adjacent layers for 3D derivatives. One of the reasons explaining the strong interest raised by Fe^II^-HCPs in the last years lies in the intrinsic porosity (or guest-induced porosity) offered by their networks and the possibility of decorating their pores through chemical functionalization. This feature, which has been studied mainly for 3D systems because of their permanent porosity, permits the uptake of a high variety of guest molecules, which, in turn, are capable of modulating the SCO properties of the HCP.^15,16^ The mechanism by which the SCO is modulated through a guest molecule depends on the nature of the interactions established between the host framework and adsorbed molecule. For example, trapped bulky guests in the 3D Fe^II^-HCP {Fe(Pz)[Pt(CN)_4_]} (Pz = pyrazine) generate steric hindrance, tending to stabilize the HS state.^17^ Conversely, other specific host–guest interactions operating in the same framework lead to stabilization of the LS state, likely due to indirect modification of the ligand-field strength of the Fe^II^ center.

Because of their interdigitated nature and therefore the lack of porosity, guest effect studies on 2D Fe^II^-HCP SCO systems are scarce and rather limited to the inclusion of small guest molecules such as water (H_2_O) or ethanol (EtOH). Often, the presence of these guests provokes elastic frustration, namely, the hindering of the metal–organic framework’s natural expansion–contraction during the HS ↔ LS transition, which usually is reflected on a decrease of the SCO temperature and/or multistepped SCO behavior.^18−27^ An increase of the SCO temperature upon H_2_O adsorption was only observed in compound {Fe(thtrz)_2_[Pd(CN)_4_]} and attributed to modification of the ligand-field strength of Fe^II^ via host–guest interactions.^18^ Recently, SCO modulation of the flexible 2D Fe^II^-HCP {Fe(5-NH_2_Pym)_2_[M^II^(CN)_4_]} (5-NH_2_Pym = 5-aminopyrimidine; M^II^ = Pt, Pd) mediated by the adsorption of H_2_O, methanol (MeOH), or EtOH was reported and justified by both electronic and steric effects operating between the protic guest molecules and 5-NH_2_Pym axial ligands.^28^ A similar guest effect was found in the related family of 2D porous compounds{Fe^II^(NCS)_2_(L)_2_}·guest [L = 1,2-bis(4′-pyridyl)ethene (tvp),^29^ 4,4′-azopyridine (azpy),^30^ 2,3-bis(4′-pyridyl)-2,3-butanediol (bpbd),^31,32^ 1,2-bis(4′-pyridyl)-1,2-ethanediol (bped),^33^ 1,2-bis(4′pyridyl)ethane (bpe);^34^ guest = acetonitrile, acetone, MeOH, EtOH, and 1-propanol], whose modifications of the SCO temperature and hysteresis width were related to specific host–guest interactions involving the L bridging ligand. All of these results indicate that functionalization of the host framework is crucial not only to promoting the binding of the guest molecule to the host framework but also to modifying through this interaction the SCO characteristics in a controlled manner.

**Scheme 1:**
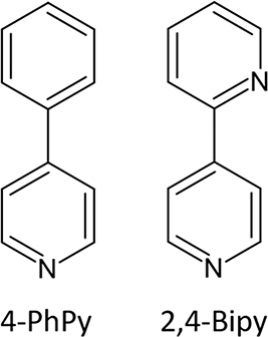
Ligands Employed in This Work: 4-PhPy and 2,4-Bipy

Aiming at providing new insights regarding the influence of host–guest interactions over the SCO behavior, here we report on a comparative study of two closely related series of 2D Fe^II^-HCPs generically formulated as {Fe^II^(L)_2_[M^II^(CN)_4_]·*n*G, with L = 4-phenylpyridine (4-PhPy) or 2,4-bipyridine (2,4-Bipy) (Scheme 1), M^II^ = Pd or Pt, and G = H_2_O and/or MeOH. Both series differ from each other by the presence or absence of a peripheral noncoordinated N heteroatom in the ancillary aromatic ring (phenyl or 2-pyridine). Although the SCO properties of the unsolvated 4-PhPy derivatives were investigated in a precedent work,^35^ their structures and foreseeable structural changes stemming from the presence/absence of guests as well as their correlation with the SCO properties have remained unknown so far. Consequently, here we analyze the structural, magnetic, and guest adsorption properties of the unsolvated and solvated forms of both series of compounds. Our results show the following: (i) from ab initio X-ray structural determinations, the unsolvated {Fe^II^(4-PhPy)_2_[M^II^(CN)_4_] (**PhPyM**; M = Pd, Pt) and {Fe^II^(2,4-Bipy)_2_[M^II^(CN)_4_] (**BipyM**; M = Pd, Pt) forms are isostructural; (ii) the presence of the N heteroatom in **BipyM** enhances considerably its sensing properties with respect to those of **PhPyM** because of the generation of stronger intermolecular interactions with the adsorbed guest molecules; (iii) single-crystal analyses of the corresponding solvated forms for both series of compounds show the occurrence of noticeable structural modifications, which, in turn, have a remarkable impact over the SCO behavior.

## Experimental section

### Synthesis

The 4-PhPy and 2,4-Bipy ligands are commercially available and were purchased from Acros Organics and Apollo Scientific, respectively.

Single crystals of **PhPyM·*x*MeOH·*y*H**_**2**_**O** (M = Pd, Pt) were obtained by slow diffusion techniques. A solution of Fe(BF_4_)_2_·6H_2_O (33.8 mg, 0.1 mmol) and the 4-PhPy ligand (31.0 mg, 0.2 mmol) in 2 mL of H_2_O/MeOH (2:1) was placed in one side of an H-shaped vessel, whereas a solution of K_2_[M(CN)_4_] [M = Pd (28.9 mg, 0.1 mmol), Pt (43.1 mg, 0.1 mmol)] in 2 mL of H_2_O was placed in the other side. Finally, the vessel was filled with a mixture of H_2_O/MeOH (1:1) and sealed with parafilm. Yellow square-plate single crystals were obtained in 1 week with a yield of ca. 50%. Anal. Calcd for **PhPyPt** [C_26_H_18_FeN_6_Pt (665.4)]: C, 46.93; H, 2.73; N, 12.63. Found: C, 46.24; H, 2.81; N, 12.42. Anal. Calcd for **PhPyPd** [C_26_H_18_FeN_6_Pd (576.7)]: C, 54.15; H, 3.15; N, 14.57. Found: C, 53.23; H, 3.26; N, 14.31.

Single crystals of **BipyM·H**_**2**_**O** (M = Pd, Pt) were obtained by slow diffusion techniques. A solution of Fe(BF_4_)_2_·6H_2_O (33.8 mg, 0.1 mmol) and the 2,4-Bipy ligand (31.2 mg, 0.2 mmol) in 2 mL of H_2_O/MeOH (3:1) was placed in one side of a H-shaped vessel, whereas a solution of K_2_M(CN)_4_ [M = Pd (28.9 mg, 0.1 mmol), Pt (43.1 mg, 0.1 mmol)] in 2 mL of H_2_O was placed in the other side. Finally, the vessel was filled with a mixture of H_2_O/MeOH (1:1) and sealed with parafilm. Yellow square-plate single crystals were obtained in 1 week with a yield ca. 50%. Anal. Calcd for **BipyPt·H**_**2**_**O** [C_24_H_18_FeN_8_OPt (685.4)]: C, 42.06; H, 2.65; N, 16.35. Found: C, 41.88; H, 2.51; N, 16.48. Anal. Calcd for **BipyPd·H**_**2**_**O** [C_24_H_18_FeN_8_OPd (596.7)]: C, 48.31; H, 3.04; N, 18.78. Found: C, 48.49; H, 2.92; N, 19.02.

### Physical Measurements

#### Magnetic Measurements

Variable-temperature magnetic susceptibility data were recorded with a Quantum Design MPMS2 SQUID magnetometer equipped with a 7 T magnet, operating at 1 T and at temperatures 50–400 K using a scan rate of 2 K min^–1^. Experimental susceptibilities were corrected for diamagnetism of the constituent atoms using Pascal’s constants.

#### Calorimetric measurements

 were performed using a Mettler Toledo DSC 821e differential scanning calorimeter. Low temperatures were obtained with an aluminum block attached to the sample holder, refrigerated with a flow of liquid nitrogen, and stabilized at a temperature of 110 K. The sample holder was kept in a drybox under a flow of dry nitrogen gas to avoid H_2_O condensation. The measurements were carried out using around 15 mg of a microcrystalline sample sealed in aluminum pans with a mechanical crimp. Temperature and heat-flow calibrations were made with standard samples of indium by using its melting transition (429.6 K; 28.45 J g^–1^). An overall accuracy of ±0.2 K in the temperature and ±2% in the heat capacity is estimated. The uncertainty increases for determination of the anomalous enthalpy and entropy due to the subtraction of an unknown baseline.

#### Single-Crystal X-ray Diffraction

Single-crystal X-ray diffraction data were collected on an Oxford Diffraction Supernova diffractometer using graphite-monochromated Mo Kα radiation (λ = 0.71073 Å). A multiscan absorption correction was performed. The structures were solved by direct methods using *SHELXS-2014* and refined by full-matrix least squares on *F*^2^ using *SHELXL-2014*.^36^ Non-H atoms were refined anisotropically, and H atoms were placed in calculated positions refined using idealized geometries (riding model) and assigned to fixed isotropic displacement parameters. All details can be found in CCDC 2090594 (**BipyPt·H**_**2**_**O**_120 K), 2090595 (**BipyPd·H**_**2**_**O**), 2090596 (**PhPyPd·MeOH·0.5H**_**2**_**O**), 2090599 (**BipyPt·H**_**2**_**O·MeOH**), 2090600 (**PhPyPd·MeOH·0.5H**_**2**_**O**), and 2090601 (**BipyPt·H**_**2**_**O**_283 K), which contain the supplementary crystallographic data for this paper.

#### Powder X-ray Diffraction (PXRD)

Measurements were performed on a PANalytical Empyrean powder X-ray diffractometer (monochromatic Cu Kα radiation) in capillary measurement mode. Because of the spontaneous rehydration of **BipyPt** and **BipyPd**, these samples were prepared by heating the hydrated forms into open capillaries inside an oven at 120 °C for 1 h and rapidly sealing them to keep air from entering. Crystal structures of compounds **PhPyM** and **BipyM** were solved ab initio using the *Topas Academic v6* program (http://www.topas-academic.net/). Very similar monoclinic unit cell parameters were found for both cases, and the atomic positions of the Pt and Fe atoms were located using the charge-flipping method.^37^ Subsequent difference Fourier maps showed the missing electron densities for the cyanide and organic ligand, which were located at the expected positions (using the corresponding solvated crystal structures as description models). The final Rietveld^38^ refinements, which showed excellent agreement between the calculated and experimental patterns, included restraints on the Pt/Fe–cyanide and Fe–organic ligand distances; the organic ligand was described by applying a semirigid body description. All details can be found in CCDC 2090597 (**PhPyPt**) and 2090598 (**BipyPt**).

#### Elemental Analyses

C, H, and N analyses were performed with a CE Instruments EA 1110 CHNS elemental analyzer.

#### Thermogravimetric analysis (TGA) experiments

 were carried out with a TA Instruments TGA550 device equipped with a Pt/Rh oven (*T*_max_ = 1000 °C). The time-dependent TGA experiments were performed by connecting the TGA apparatus to a flow mass controller. Thus, humid air was passed at room pressure and a temperature of 30 °C and driven into the TGA chamber, where a previously desolvated sample of **BipyPt** or **BipyPd** was mounted in a Pt pan.

## Results

### Synthesis and Chemical Characterization

Single crystals of **PhPyM·*x*MeOH·*y*H**_**2**_**O** and **BipyM·H**_**2**_**O** (M = Pt, Pd) were grown by liquid–liquid slow diffusion using H-shaped tubes containing, on the one side, a H_2_O/MeOH solution of a Fe^II^-4-PhPy or -2,4-Bipy mixture and, on the other side, an aqueous solution of the corresponding [M(CN)_4_]^2–^ potassium salt (see the Experimental Section for more details).

PXRD (Figure S1) and magnetic measurements (vide infra) of pristine crystals of **PhPyM·*x*MeOH·*y*H**_**2**_**O** soaked in the mother liquor indicate that the synthesis method affords an imprecise mixture of solvates with *x* and *y* in the intervals 0–1 and 0–0.5, respectively. Only the crystal structure of the majority component of these solvates was successfully identified and presents the formula **PhPyM·MeOH·0.5H**_**2**_**O**. Once removed from the mother liquor, crystals of **PhPyM·*x*MeOH·*y*H**_**2**_**O** spontaneously desorb most of the guest molecules, provoking a total loss of crystallinity. This was confirmed by elemental analysis, PXRD, TGA (see the Experimental Section and Figures S1 and S2), and magnetic measurements (vide infra). Indeed, according to TGA, only an equivalent weight of 0.2–0.4 molecules of H_2_O remains in the dried samples at ambient conditions. Besides, solvent-free **PhPyM** derivatives are easily obtained from a thermal treatment at 400 K for 10 min. In view of the changes detected in the PXRD patterns upon solvent desorption (Figure S1), the loss of guest molecules is accompanied by relevant structural changes.

It is worth noting that **PhPyM** does not readsorb H_2_O from air moisture. This was deduced from TGA measurements performed on desorbed **PhPyM** samples exposed to air for several days, which showed no mass loss up to 530 K when the structures start to decompose (Figure S2).

Chemical characterization of **BipyM·H**_**2**_**O** (M = Pt, Pd) shows that the guest H_2_O molecule is retained within the structure once the samples are removed from the mother liquor and exposed at ambient conditions (see elemental analysis, PXRD patterns, and TGA measurements in the Experimental Section and Figures S3 and S4, respectively). Nonetheless, according to the TGA measurements (Figure S4), the H_2_O molecule can be easily desorbed with gentle heating above 300 K, giving rise to the dehydrated **BipyM** counterparts. Similar to that observed in **PhPyM**, H_2_O desorption provokes relevant structural modifications in view of the differences detected from the PXRD patterns of the solvated and desolvated compounds (Figure S3).

**Scheme 2:**
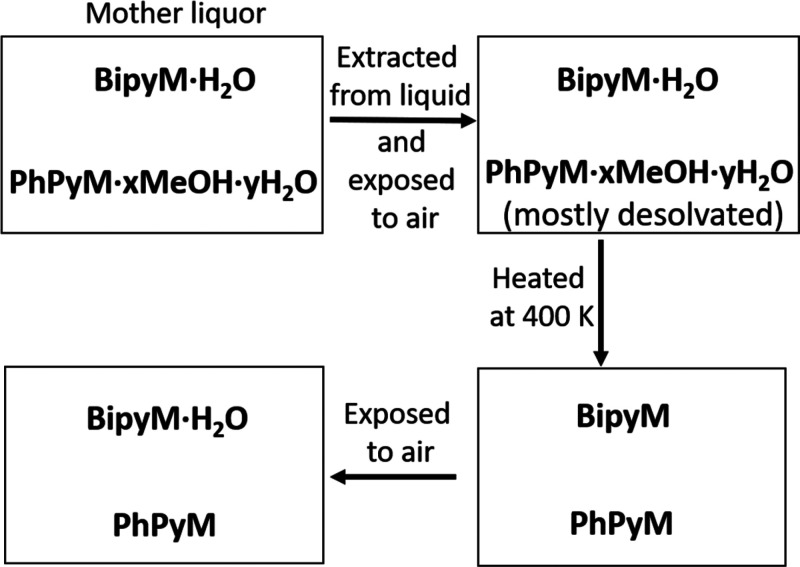
Adsorption and Desorption Processes Observed for Compounds **PhPyM·*x*MeOH·*y*H**_**2**_**O** and **BipyM·H**_**2**_**O** upon Different Treatments

TGA studies show, in contrast to that observed for **PhPyM**, that both **BipyPt** and **BipyPd** are capable of gradually readsorbing one molecule of H_2_O per Fe^II^ ion from air moisture. Indeed, the H_2_O readsorption monitored in situ by TGA reveals that **BipyM** recovers the original monohydrated phase in less than 1 h (see Scheme 2 and Figure S5). Importantly, the close similarity between the PXRD patterns of compounds **PhPyM** and **BipyM** (Figure S6) points out that the corresponding desorbed phases are isostructural.

### Magnetic Characterization

#### SCO Properties of **PhPyM·*x*MeOH·*y*H**_**2**_**O** and **BipyM·H**_**2**_**O** (M = Pt, Pd)

**Figure 1:**
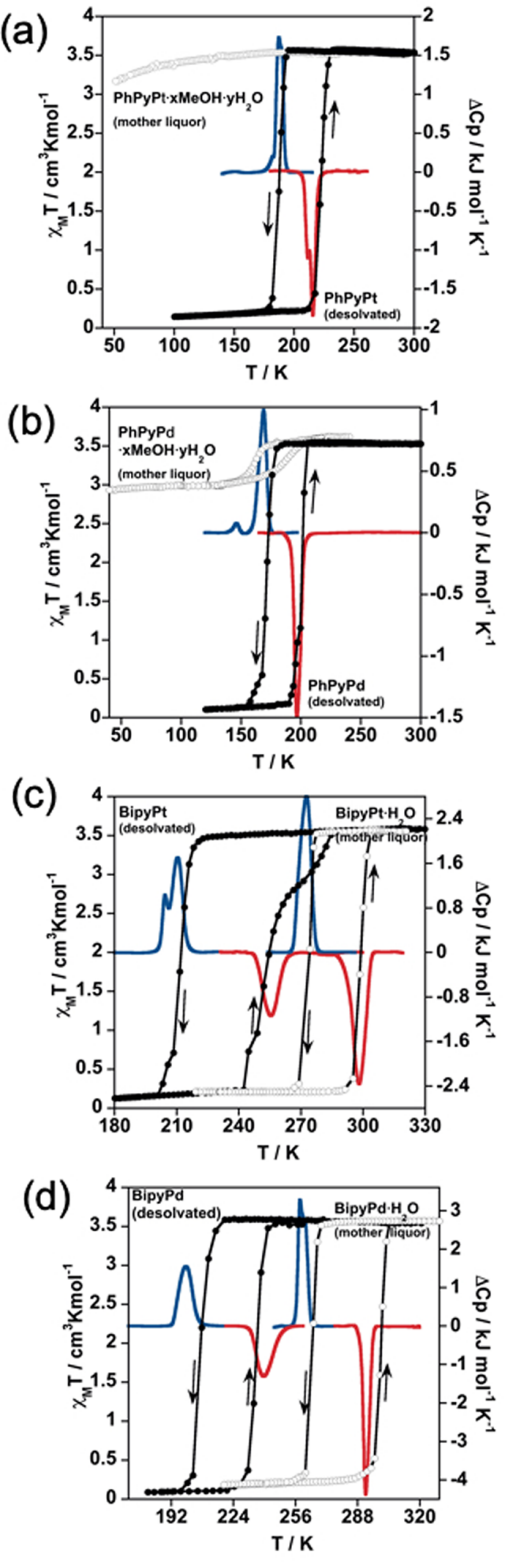
Thermal dependence of χ_M_*T* for: (i) **PhPyM·*x*MeOH·*y*H**_**2**_**O** [M = Pt (a), Pd (b)] measured in the mother liquor (open circles) and after treatment at 400 K for 1 h (filled circles) and (ii) **BipyM·H**_**2**_**O** [M = Pt (c), Pd (d)] measured in the mother liquor (open circles) and after treatment at 400 K for 1 h (filled circles). Calorimetric measurements in the cooling (blue curves) and heating (red curves) modes of the desolvated counterparts of **PhPyPt** (a), **PhPyPd** (b), **BipyPt** (c), and **BipyPd** (d) and the hydrated air-dried products of **BipyPt·H**_**2**_**O** (c) and **BipyPd·H**_**2**_**O** (d).

Figure 1 displays the thermal dependence of χ_M_*T* (where χ_M_ is the molar magnetic susceptibility and *T* is the temperature) for **PhPyM·*x*MeOH·*y*H**_**2**_**O** and **BipyM·H**_**2**_**O** and for their respective complete desolvated forms (χ_M_*T* vs *T* plots of the partially desolvated **PhPyM·*x*MeOH·*y*H**_**2**_**O** forms are shown in Figure S7). In order to monitor the SCO behavior of the pristine solvated forms and considering their propensity to lose the included solvent molecules, the magnetic properties were measured soaked in their mother liquor (open circles in Figure 1a,b). At 240 K, χ_M_*T* is ca. 3.5 cm^3^ K mol^–1^ for compound **PhPyPt·*x*MeOH·*y*H**_**2**_**O**, which is assignable to a fully HS (*S* = 2) Fe^II^ ion (Figure 1a). This value remains practically constant within the temperature range 240–50 K, proving that this compound does not exhibit SCO properties. Similarly, compound **PhPyPd·*x*MeOH·*y*H**_**2**_**O** shows a χ_M_*T* value of 3.5 cm^3^ K mol^–1^ between 300 and 170 K (Figure 1b). However, below this temperature, χ_M_*T* decreases abruptly by 0.5 cm^3^ K mol^–1^ (*T*_1/2_^↓^ = 164 K), denoting the occurrence of a HS-to-LS transition involving ca. 14% of the Fe^II^ centers. In the heating mode, the χ_M_*T* versus *T* plot does not match the cooling mode, with the latter being more gradual and shifted to higher temperatures (*T*_1/2_^↑^ = 185 K), thereby defining an asymmetric hysteresis loop with *T*_1/2_ = 174.5 K and Δ*T* = 21 K [*T*_1/2_^↓^/*T*_1/2_^↑^ are the equilibrium temperatures at which 50% of the SCO-active Fe^II^ ions have changed from spin state during the cooling/heating modes, *T*_1/2_ = (*T*_1/2_^↑^ + *T*_1/2_^↓^)/2, and Δ*T* is the hysteresis width *T*_1/2_^↑^ – *T*_1/2_^↓^].

As was already mentioned, removing crystals of **PhPyM·*x*MeOH·*y*H**_**2**_**O** (M = Pt, Pd) from their mother liquor provokes instantaneous loss of most of the guest molecules (Figure S2), causing important modifications in the magnetic properties of both the Pt and Pd derivatives. Indeed, the SCO of both air-dried samples defines an almost complete and asymmetric hysteresis loop characterized by a double step in the cooling mode (*T*_1/2_^↓1^ = 202 K and *T*_1/2_^↓2^ = 180 K for Pt; *T*_1/2_^↓1^ = 182 K and *T*_1/2_^↓2^ = 163 K for Pd) and a single step in the heating mode (*T*_1/2_^↑^ = 218 K, Δ*T*^1^ = 16 K, and Δ*T*^2^ = 38 K for Pt; *T*_1/2_^↑^ = 200 K, Δ*T*^1^ = 18 K, and Δ*T*^2^ = 37 K for Pt) (Figure S7). The subsequent treatment at 400 K for 1 h leads to the completely desorbed forms **PhPyPt** and **PhPyPd**, which exhibit abrupt, complete, and hysteretic one-step spin transitions with *T*_1/2_/Δ*T* of 204/36 K and 185/29 K, respectively (filled circles in Figure 1a,b). These curves were perfectly reproduced for the same samples several days after exposure to ambient conditions (Figure S8), confirming, in good agreement with the TGA studies (Figure S2), that these compounds are not prone to readsorb H_2_O from air moisture.

Freshly prepared soaked crystals of **BipyM·H**_**2**_**O** (M = Pt, Pd) exhibit abrupt, complete, and hysteretic spin transitions centered at room temperature with *T*_1/2_/Δ*T* of 286.5/25 K and 282.5/35 K, respectively (open circles in Figure 1c,d). According to the TGA data (Figure S4), the H_2_O molecule included in **BipyPt·H**_**2**_**O** and **BipyPd·H**_**2**_**O** is retained at *T* ≤ 300 K in contact with air. Consequently, to ensure the retention of H_2_O and considering the dry atmosphere and vacuum conditions of the SQUID chamber, the magnetic properties of the air-dried samples were checked first in the temperature sequence 290–220 K in order to compare them with those of the soaked samples. As expected, the SCO curves recorded upon cooling [*T*_1/2_^↓^ = 272 K (Pt) and 273 K (Pd); Figure S9] are close to those obtained for the crystals soaked in the mother liquor [275 K (Pt) and 266 K (Pd)], confirming that the H_2_O molecule persists within the structure at ambient conditions. In order to analyze the heating branch, χ_M_*T* was measured in the temperature range 220–320 K, obtaining *T*_1/2_^↑^ values of 303 K (Pt) and 293 K (Pd). The resulting SCO curves are very similar to those of the soaked samples (Figure S9), suggesting that, although the solvates can be subjected to dehydration during the process of heating above 300 K, either the effective desolvation must be very small or it does not affect the *T*_1/2_^↑^ value. When compounds **BipyPt·H**_**2**_**O**/**BipyPd·H**_**2**_**O** are heated at 400 K for 1 h inside the SQUID magnetometer, the H_2_O molecule is totally evacuated, affording the desorbed counterparts **BipyPt**/**BipyPd**. The dehydrated derivatives also display strongly cooperative SCO behaviors (filled circles in Figure 1c,d) but dramatically downshifted in temperature by 54 K (Pt) and 61.5 K (Pd) with respect to their corresponding hydrated counterparts. Nevertheless, the hysteresis widths are overall maintained (Δ*T* = 41/28 K for **BipyPt**/**BipyPd**). In good agreement with the TGA measurements, the room temperature centered SCO curves of the original monohydrated derivatives are completely recovered when the desorbed samples are exposed to air for ca. 1 h (Figure S10).

**Figure 2:**
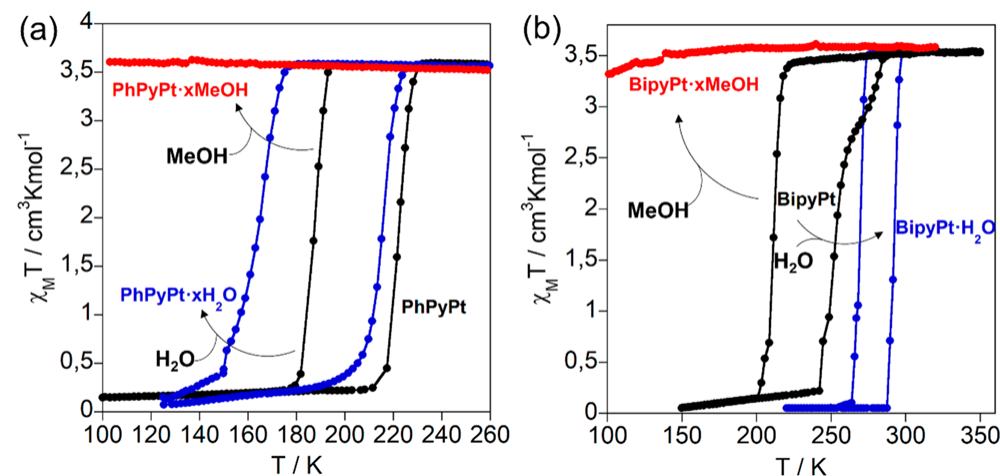
SCO properties of (a) **PhPyPt** and (b) **BipyPt** before (black curves) and after adsorption of H_2_O (blue curves) and MeOH (red curves).

In order to investigate the capability of the unsolvated samples to reinclude MeOH or H_2_O molecules in the structure and to assess the degree of reversibility of the magnetic behavior described above, the desolvated **PhPyM** and **BipyM** derivatives were dispersed in MeOH or H_2_O for several hours. In order to evaluate the eventual structural changes associated with the adsorption processes, PXRD patterns were performed for the solids immersed in the corresponding solvents (Figures S11 and S12). The results show the occurrence of important modifications in the patterns of both **PhPyM** and **BipyM** when they are soaked in MeOH. In particular, the shift of the 002 peak centered around 7.6° toward lower 2θ values reflects an increase in separation between two consecutive bimetallic layers as a consequence of inclusion of the MeOH molecule. Besides, whereas noticeable modifications of the patterns of **BipyM** soaked in H_2_O confirm the adsorption of H_2_O in this network, patterns of **PhPyM** are virtually unchanged in H_2_O, indicating a negligible amount of adsorbed H_2_O. The χ_M_*T* versus *T* curves of the soaked crystals (Figures 2 and S13 for Pt and Pd derivatives, respectively) clearly reveal that the adsorption of MeOH stabilizes the HS state at all temperatures for the four compounds (black curves in Figures 2 and S13). In contrast, the adsorption of H_2_O in **PhPyM** and **BipyM** provokes opposite effects on their respective spin transitions. For **PhPyM**, the average *T*_1/2_ values slightly decrease from 204 to 190 K (M = Pt) and from 185 to 180 K (M = Pd), and the hysteresis width Δ*T* increases from 35 to 51 K (M = Pt) and from 29 to 36 K (M = Pd). For **BipyM**, their *T*_1/2_ values increase considerably from 238 to 281.5 K (M = Pt) and from 221 to 282 K (M = Pd), while Δ*T* decreases from 32 to 23 K (M = Pt) and remains unchanged for M = Pd. Importantly, magnetic (Figure S14) and TGA (Figure S15) characterizations performed for compounds **PhPyM·*x*solv** (solv = H_2_O, MeOH) and **BipyM·*x*MeOH** a few minutes after removing them from the corresponding solvent clearly suggest the occurrence of rapid desorption of the guest molecules, a fact that avoids a proper estimation of *x*. In contrast, as aforementioned, the air-dried samples of **BipyM·H**_**2**_**O** (M = Pt^II^, Pd^II^) maintain the H_2_O molecule per Fe^II^ ion of the initial as-synthesized hydrated framework.

### Included H_2_O-Dependent SCO in **BipyPt·*x*H**_**2**_**O** (*x* = 0–1)

**Figure 3:**
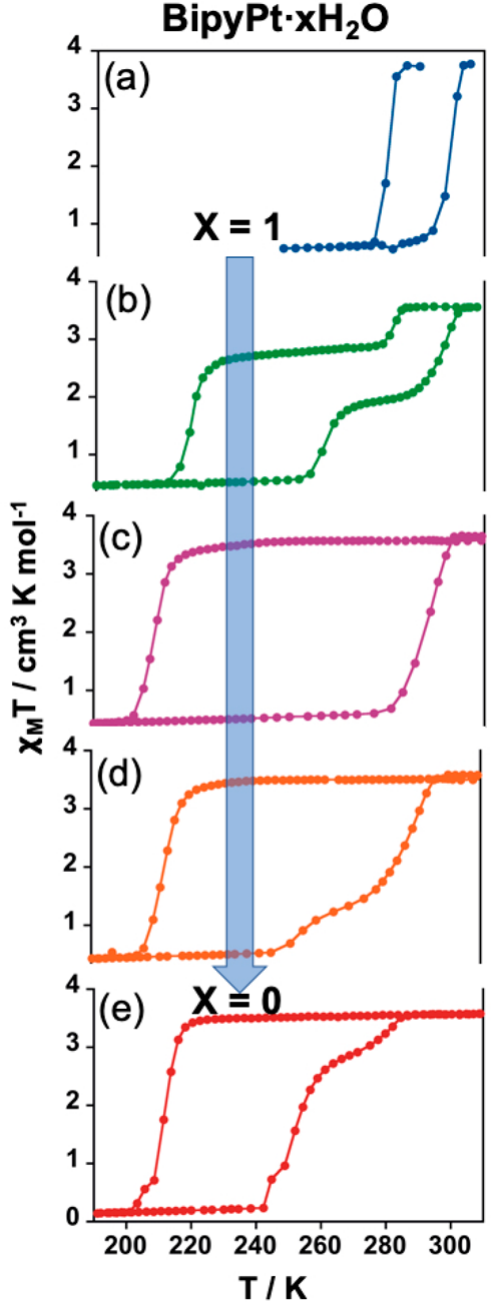
Evolution of the SCO properties of **BipyPt·*x*H**_**2**_**O** from the (a) fully hydrated (*x* = 1) to the (e) completely dehydrated (*x* = 0) derivatives. χ_M_*T* versus *T* curves displayed in parts b–d were obtained by the in situ sequential heating of the original hydrated sample (see the text).

The marked difference observed between the SCO temperatures of the dehydrated (*x* = 0) and hydrated (*x* = 1) counterparts in the **BipyM·*x*H**_**2**_**O** systems (Figure 2b) encouraged us to investigate the SCO profiles of some intermediate degrees of hydration. Even if we were unable to estimate the exact amount of H_2_O for these intermediate hydrates, we managed to get hydration degrees from *x* = 1 to 0 by the sequential controlled heating of a pristine hydrated sample of **BipyPt·H**_**2**_**O** inside the SQUID magnetometer. First, a fresh **BipyPt·H**_**2**_**O** sample was measured in the 290–180–305 K temperature sequence, obtaining the expected SCO behavior of the completely hydrated framework (Figure 3a). Then, the solid was heated up to 305 K for 10 min and the thermal variation of χ_M_T subsequently registered in the 305–180–320 K temperature range. The resulting curve shows the split of the SCO in two defined steps with *T*_1/2_^↓1^/*T*_1/2_^↑1^ = 278/298 K and *T*_1/2_^↓2^/*T*_1/2_^↑2^ = 220/264 K, which are reminiscent of the SCO behaviors of the completely hydrated and dehydrated compounds, respectively (Figure 3b). After that, the sample was heated at 320 K for 10 min followed by monitoring of the χ_M_*T* values with the 320–180–320 K temperature range. Surprisingly, the SCO curve registered after this thermal treatment exhibits an outstanding hysteresis loop with a Δ*T* value of 84 K and values of *T*_1/2_^↓^ and *T*_1/2_^↑^ of 208 and 292 K, respectively (Figure 3c). Hence, whereas *T*_1/2_^↓^ is comparable to that of the dehydrated compound, *T*_1/2_^↑^ is very similar to that of the hydrated counterpart. Afterward, the sample was heated again at 320 K and measured within the same temperature range, observing an exclusive modification of the heating branch, which reflects the apparition of two steps (*T*_1/2_^↑2^ = 256 K and *T*_1/2_^↑1^ = 286 K), therefore delineating an asymmetric hysteresis loop (Figure 3d). Finally, subsequent treatment at 400 K led to the above-mentioned SCO behavior of the dehydrated compound (Figure 3e).

### Calorimetric Measurements

Differential scanning calorimetry analysis was performed in order to confirm the SCO behaviors obtained by the SQUID measurements. The calorimetry data of desolvated **PhPyPt**, **PhPyPd**, **BipyPt**, and **BipyPd** and hydrated **BipyPt·H**_**2**_**O** and **BipyPd·H**_**2**_**O** compounds were conducted with a scan rate of 10 K min^–1^ in the cooling and heating modes, obtaining the corresponding Δ*C*_p_ versus *T* curves displayed in Figure 1. The *T*_1/2_ and Δ*T* values extracted from these curves (Table 1) are in very good accord with the magnetic data. The slight discrepancies obtained (notably in the *T*_1/2_^↓^ values) are related to the different scan rates used for the different techniques. Furthermore, the double peaks observed for some of the samples reflect subtle changes of the slope of the γ_HS_ vs T curve during the HS–LS transformation and usually are associated with structural rearrangements concomitant with the spin transition. The estimated average Δ*H* and Δ*S* variations involved in the spin-state changes (Table 1) are comparable with those observed for cooperative SCO of related 2D Hofmann-type clathrates.^11−13^ The Δ*C*_p_(*T*) curve of an intermediate hydrated state was also registered after gentle heating of compound **BipyPt·H**_**2**_**O** inside the calorimeter device. As a result, the cooling and heating Δ*C*_p_ versus *T* curves reflect a double-step SCO consistent with that observed in the magnetic study (Figure S16). The average Δ*H* (kJ mol^–1^)/Δ*S* (J mol^–1^ K^–1^) values associated with the first and second steps are 6.565/28.484 and 7.058/24.626, respectively.

**Table 1:** Thermodynamic Parameters Extracted from the Magnetic and Calorimetric Measurements

	magnetism		calorimetry			
compound	*T*_1/2_ (K)	Δ*T* (K)	*T*_1/2_ (K)	Δ*T* (K)	Δ*H* (kJ mol^–1^)	Δ*S* (J mol^–1^ K^–1^)
**BipyPt**	238	32	233	44	13.23	63.31
BipyPt·H_**2**_**O**	286.5	25	285	24	17.99	63.22
**BipyPd**	221	28	219.5	39	12.90	59.15
BipyPd·H_**2**_**O**	282.5	35	273.5	37	16.57	61.75
**PhPyPt·*x*MeOH·*y*H**_**2**_**O**	no SCO					
**PhPyPt**	204	36	201.5	29	10.29	47.98
**PhPyPd·*x*MeOH·*y*H**_**2**_**O**	174.5 (incomplete)	21				
**PhPyPd**	185	29	181	28.5	11.41	57.09

### Crystal Structures

Crystal structures of **PhPyM·MeOH·0.5H**_**2**_**O** (M = Pt, Pd) were successfully obtained at 120 K (HS), while that of **BipyPt·H**_**2**_**O** was measured at 283 K (HS) and 120 K (LS). However, the structure of **BipyPd·H**_**2**_**O** was analyzed only at 120 K (LS) because these crystals rapidly lose their crystallinity when measured above 280 K. Furthermore, the structure of compound **BipyPt·H**_**2**_**O·MeOH**, obtained by soaking crystals of **BipyPt·H**_**2**_**O** in pure MeOH for several days, was determined at 120 K (HS). The crystal structures of the unsolvated **PhPyPt** and **BipyPt** counterparts were solved at 298 K (HS) from ab initio methods using the *Topas Academic v6* program through the treatment and Rietveld refinement of the corresponding PXRD patterns (see the Experimental Section for more details). Relevant crystallographic data for all compounds are gathered in Tables S1–S3, while the corresponding significant metal-to-ligand bond lengths, angles, and intermolecular interactions are given in Tables S4–S6, respectively. Rietveld plots are given in Figure S17 for the final refinements.

#### Structure of **PhPyM·MeOH·0.5H**_**2**_**O** (M = Pt, Pd)

**Figure 4:**
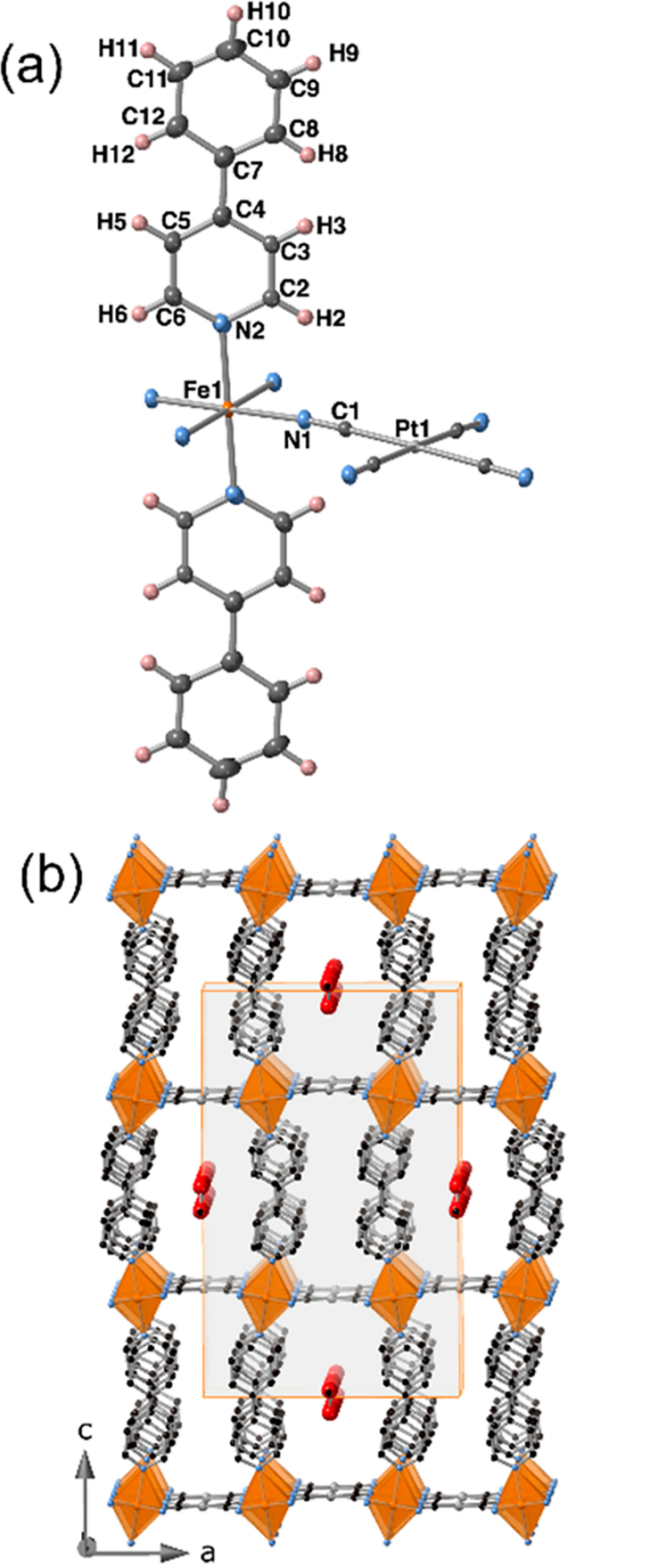
(a) ORTEP representation of the Fe^II^ environment displayed by **PhPyM·MeOH·0.5H**_**2**_**O** (M = Pt, Pd). (Atoms are represented at 50% probability). (b) Fragment of the 2D networks formed by four stacked layers showing the channels where the solvent molecules are located in **PhPyM·MeOH·0.5H**_**2**_**O**.

At 120 K, the crystal structures of **PhPyM·MeOH·0.5H**_**2**_**O** (M = Pt, Pd) were determined in the orthorhombic *Imma* space group. They consist of a crystallographic unique type of octahedral {FeN_6_} site coordinated equatorially by four N atoms belonging to four equivalent square-planar [M(CN)_4_]^2–^ units, which bridge four other equivalent Fe^II^ centers, forming bimetallic {Fe^II^[M^II^(CN)_4_]}_*n*_ layers. The axial positions are occupied by two equivalent terminal 4-phenylpyridine ligands (Figure 4a), thereby completing the 2D framework. At 120 K, the average Fe–N bond length is 2.179 and 2.181 Å for Pt and Pd derivatives, respectively, reflecting that, even at low temperature, the Fe^II^ ions are in the HS state, in good agreement with the magnetic data and the yellow color of the crystals. The {Fe^II^(4-PhPy)_2_[M^II^(CN)_4_]}_*n*_ layers are pillared in such a way that the axial 4-PhPy ligands of adjacent layers are interdigitated, establishing π–π stacking interactions where the centroid-to-centroid distances of the face-to-face aromatic rings are 3.720 Å (M^II^ = Pt) and 3.740 Å (M^II^ = Pd) (Figure S18a). Moreover, one molecule of MeOH and a half molecule of H_2_O per unit cell are hosted within the interlayer hydrophobic space, inducing a tilt of the [FeN_6_] octahedra and a slight corrugation of the bimetallic {Fe^II^[M^II^(CN)_4_]}_*n*_ planes (Figure 4b). Indeed, the [Fe(N_eq_)_4_] equatorial plane of the [FeN_6_] octahedron defines average angles of 8.6° (M^II^ = Pt) and 9.1° (M^II^ = Pd) with the square-planar units [M^II^(CN)_4_]^2–^, thereby generating by symmetry two inequivalent channels between the interdigitated layers. Whereas one of these channels does not contain any guest due to the small void left by the neighboring 4-PhPy ligands, the other is wide enough to host the solvent molecules. These guest molecules are distributed along the channel in such a way that they define a plane that is stabilized through hydrogen bonds and situated at 2.6 Å with respect to the aromatic H atoms of the 4-PhPy ligand.

#### Structure of **BipyM·H**_**2**_**O** (M = Pt, Pd)

**Figure 5:**
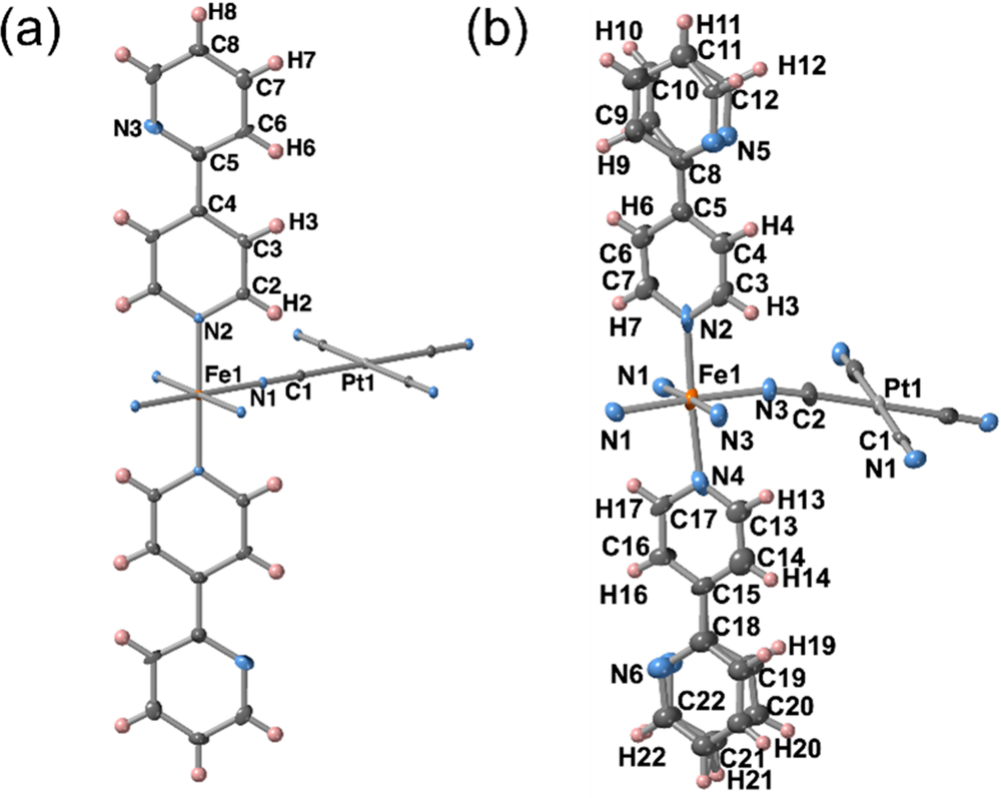
ORTEP representations of the Fe^II^ environment displayed by (a) **BipyPt·H**_**2**_**O** and (b) **BipyPt·H**_**2**_**O·MeOH**. Atoms are represented at 50% probability.

**Figure 6:**
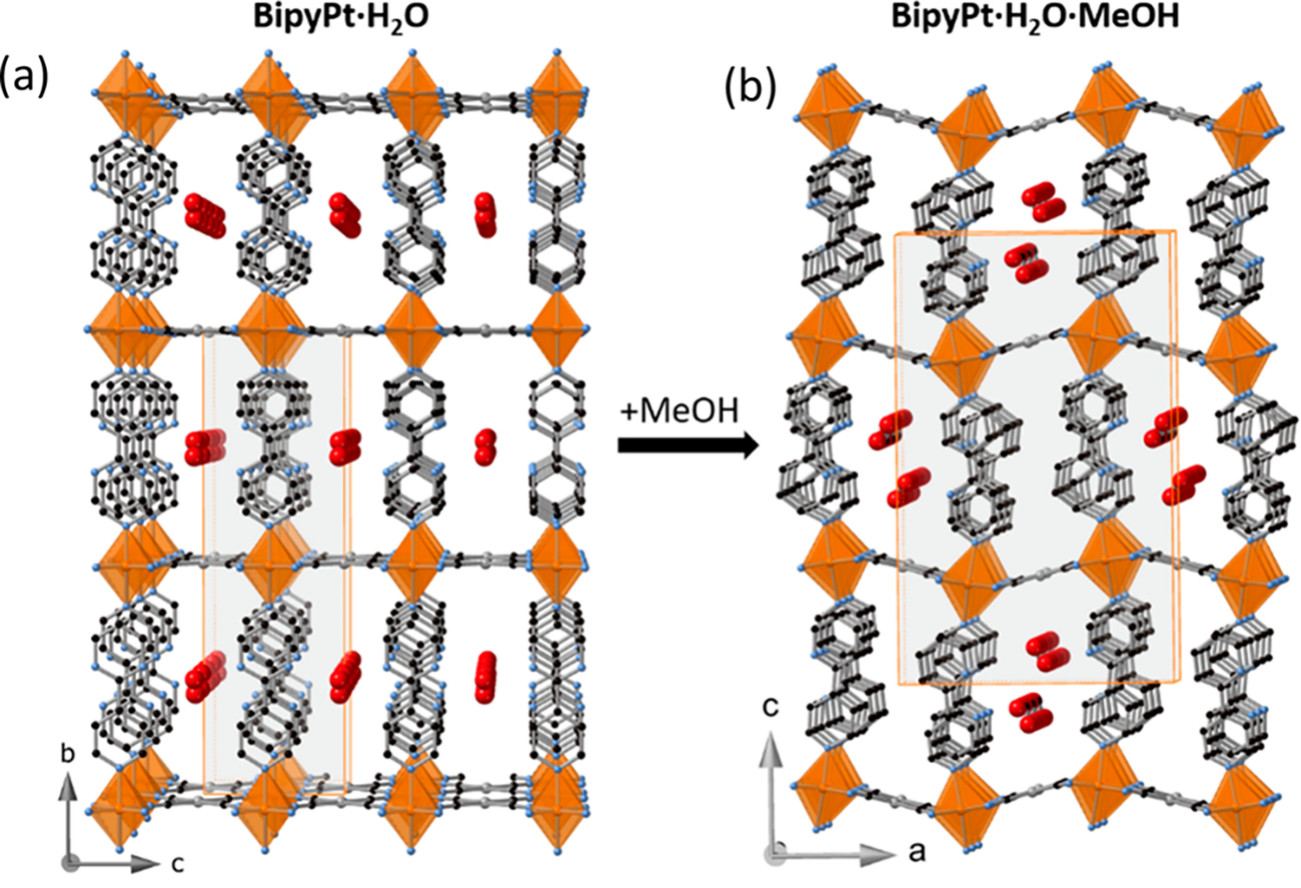
Structural modifications observed on the (a) **BipyPt·H**_**2**_**O** (isostructutal to **BipyPd·H**_**2**_**O**) network upon MeOH adsorption and the corresponding transformation in (b) **BipyPt·H**_**2**_**O·MeOH**. The two disordered positions of the noncoordinated pyridine are shown for compound **BipyPt·H**_**2**_**O·MeOH**.

At 120 K, the crystal structures of **BipyM·H**_**2**_**O** present the orthorhombic *Cmmm* space group. The structure consists of a unique octahedral {FeN_6_} site coordinated by four equivalent square-planar [M(CN)_4_]^2–^ bridging ligands in the equatorial positions and by the 4-substituted pyridine N atom of two equivalent terminal 2,4-bipy ligands in the axial positions (Figure 5a). It is worth noting that atoms N3 and C6 are disordered by symmetry and have been modeled with an occupancy of 0.5 in each position. Conversely to the 4-Phpy-based compounds, the average Fe–N distances are 1.953 and 1.958 Å for **BipyPt·H**_**2**_**O** and **BipyPd·H**_**2**_**O**, respectively, indicating that the Fe^II^ ion is in the LS state, in good agreement with the magnetic data and the red color of the crystals. Furthermore, the {Fe^II^[M^II^(CN)_4_]}_*n*_ layers are strictly planar (Figure 6a), in contrast to that observed for **PhPyM·MeOH·0.5H**_**2**_**O**. As a result of their higher structural homogeneity, only one type of channel is generated between the interdigitated bimetallic layers where one molecule of H_2_O is hosted, occupying discrete positions and interacting via moderate hydrogen bonds with the N3 heterocyclic atom of the 2,4-bipy ligand (O1···N3 intermolecular distance of 2.953 Å for Pt and 3.000 Å for Pd). Moreover, the pillared layers are stabilized by π–π interactions established between the pyridine moieties of the interdigitated 2,4-bipy ligands with centroid-to-centroid distances of 3.665 and 3.671 Å for **BipyPt·H**_**2**_**O** and **BipyPd·H**_**2**_**O**, respectively (Figure S18b). In good agreement with the magnetic data, crystals of **BipyPt·H**_**2**_**O** become yellow upon heating, evidencing the occurrence of a LS-to-HS transition. This was confirmed by analysis of the structure at 283 K, which is basically the same as that at 120 K but exhibiting, as the main difference, an increase of 0.206 Å in the Fe–N average distance.

#### Structure of **BipyPt·H**_**2**_**O·MeOH**

When crystals of **BipyPt·H**_**2**_**O** are immersed in pure MeOH for several days, they adsorb one molecule of MeOH, leading to compound **BipyPt·H**_**2**_**O·MeOH**. The MeOH uptake is accompanied by a single-crystal-to-single-crystal transformation from the orthorhombic *Cmmm* space group to the orthorhombic *Pnma* space group. This loss of symmetry is reflected in the apparition of two nonequivalent 2,4-Bipy ligands as well as two distinct types of equatorial coordinating N atoms (N1 and N3). Furthermore, the entry of MeOH induces the occurrence of a positional disorder on the noncoordinated pyridine of the 2,4-bipy ligand (Figure 5b). At 120 K, crystals of **BipyPt·H**_**2**_**O·MeOH** are yellow and consist, analogously to **BipyPt·H**_**2**_**O**, of a layered structure. However, in contrast with the monohydrated phase, the bimetallic layers are considerably corrugated likely provoked by inclusion of the molecule of MeOH, which distorts the 2D network (Figure 6b). This corrugation is reflected on the angles of 6.54° and 23.01° defined between the [Fe(N_eq_)_4_] equatorial plane of the [FeN_6_] octahedron and the adjacent square-planar units [Pt^II^(CN)_4_]^2–^. The guest MeOH molecules are accommodated along the channels generated between the protruding interdigitated 2,4-bipy axial ligands of consecutive layers, where one molecule of H_2_O has also been detected. Both guests establish hydrogen-bonding interactions (N–O distances in the 2.8–3.3 Å range) with the noncoordinated N heteroatom of the axial 2,4-bipy ligand. The Fe–N average distance is 2.160 Å, which indicates that the structure is blocked at the HS state even at 120 K.

#### Structures of **BipyPt** and **PhPyPt**

**Figure 7:**
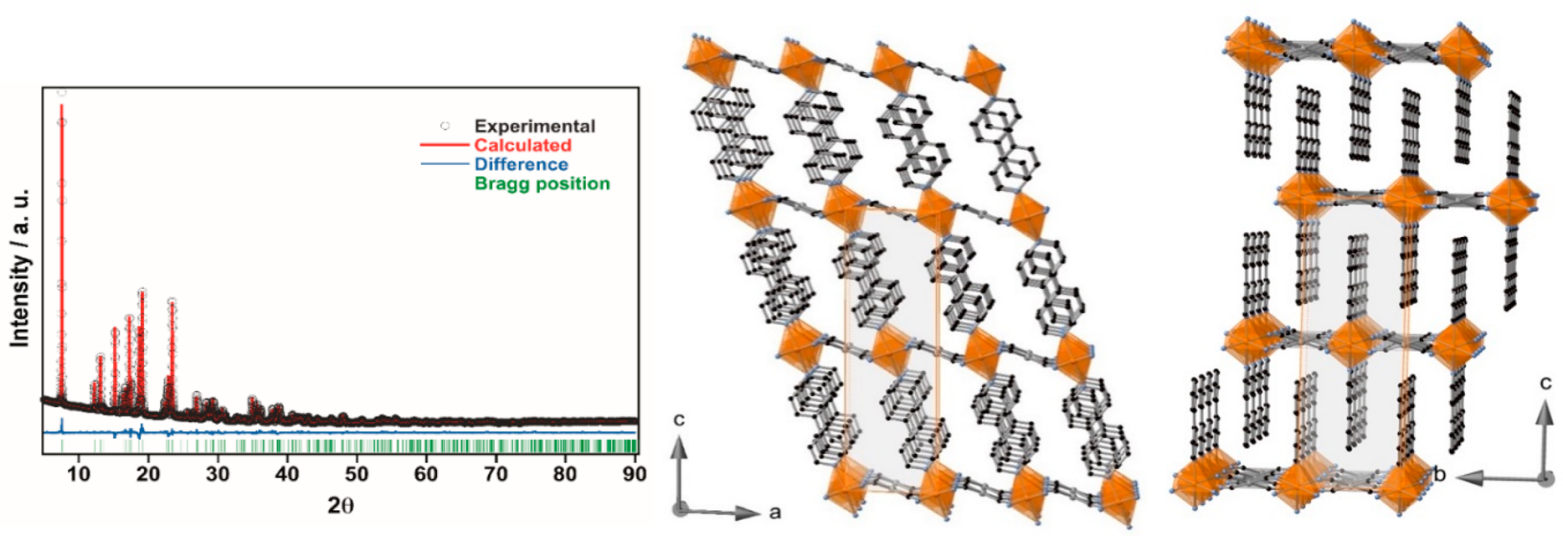
Rietveld fitting of the experimental XRPD pattern for **PhPyPt** (left) (for **BipyPt**, see Figure S17). Views along the *b* (middle) and *a* (right) axes of a fragment of the desolvated **PhPyPt** framework.

The solvent-free **BipyPt** and **PhPyPt** compounds are isostructural and adopt the monoclinic *I*2/*m* space group at 298 K. The Fe^II^ ion lies in an inversion center with average Fe–N bond lengths of 2.167 and 2.168 Å for **BipyPt** and **PhPyPt**, respectively, which are consistent with the HS state of the Fe^II^ site. Analogously to the corresponding solvated forms, the structure is composed of an infinite stack of bimetallic {Fe(L)_2_[Pt(CN)_4_]} layers. However, at variance with the previously described structures, the layers of unsolvated forms are strongly corrugated (Figure 7). Indeed, the angle defined by the average equatorial planes [Fe(N_eq_)_4_] and [Pt(CN)_4_]^2–^ is around 38° for both derivatives. This corrugation induces a marked tilt of the axial ligands with respect to the mean plane defined by the Fe^II^-M^II^ bimetallic layer favoring a considerably more dense and efficient packing between layers in such a manner that no solvent-accessible channels are present within the structures.

## Discussion

The main objective of this work was to evaluate the affinity toward adsorption of hydrophilic guests in two equivalent series of 2D Hofmann-type {Fe^II^(L)_2_[M^II^(CN)_4_]} (M^II^ = Pt^II^, Pd^II^) CPs based on the axial ligands L = 4-PhPy (**PhPyM**) and 2,4-Bipy (**BipyM**) and to assess the structural effects induced by adsorption/desorption of the guest and its consequences on the SCO properties. Both series of 2D frameworks essentially differ in the presence/absence of an extra N heteroatom and consequently in the distinct hydrophobicity of the interlayer spaces generated between the interdigitated 2,4-Bipy and 4-PhPy ligands.

### Solvent Affinity and Structural Rearrangements

The different capabilities of compounds **PhPyM** and **BipyM** for retaining MeOH or H_2_O can be rationalized by analyzing the structures of the corresponding solvates. For example, in the **PhPyM·MeOH·0.5H**_**2**_**O** and **BipyM·H**_**2**_**O·MeOH** clathrates, the interactions of the MeOH molecules with the host framework are rather repulsive because of the steric requirements created by the methyl group of the MeOH molecule. As a result, the interdigitated 4-PhPy or 2,4-bipy ligands are tilted, maximizing the host–guest distances and forcing the aperture of channels in the interlayer space to facilitate the inclusion of MeOH. However, on the basis of the magnetic behavior (Figures 2 and S13) and PXRD (Figure S11), these distorted structures are adopted when the crystals are soaked in MeOH, but they are not stable once exposed to air, thereby losing the guest molecules and reverting to the desorbed **PhPyM** and **BipyM** phases. In contrast, the H_2_O molecules located between adjacent layers in **BipyM·H**_**2**_**O** occupy discrete positions in such a way that they establish hydrogen bonds with the N heteroatom of the noncoordinating pyridine of the 2,4-bipy ligand. As a result of this hydrogen bonding, the H_2_O molecule remains attached to the host framework unless a heating treatment is applied. The H_2_O molecules are situated at the center of the square windows defined by four neighboring interdigitated 2,4-bipy ligands and the equatorial CN^–^ groups. Hence, their presence does not suppose any steric repulsion over the host network, promoting a very regular, ordered, and stable structure reminiscent of those of the analogues 2D-Hofmann compounds {Fe(pyrimidine)_2_[M(CN)_4_]}·*x*H_2_O (**PymM**)^39^ and {Fe(pyridazine)_2[_M(CN)_4_]·*x*H_2_O} (**PdzM**).^24^

Whereas desorbed compounds **PhPyM** are not capable of recovering H_2_O from humid air, compounds **BipyM** exhibit a strong affinity to H_2_O, readsorbing it spontaneously within a few minutes. It is important to stress that this marked difference cannot be ascribed to structural factors because both unsolvated forms are isostructural and isomorphous; consequently, the interstitial space generated within the interdigitated axial ligands of two consecutive layers is similar in both series of compounds. Indeed, the presence of the N heteroatom in **BipyM** is the driving force responsible for its high affinity toward H_2_O, which is sequestered by the host framework, triggering a crystal transformation. Thus, crystals convert from the desorbed phase **BipyM** (monoclinic *C*) to the monohydrated phase **BipyM·H**_**2**_**O** (orthorhombic *C*), inducing a large “wine-rack”-like transformation to accommodate the H_2_O molecules. These marked structural changes are unprecedented within the family of 2D Hofmann-type frameworks, which show the ability to recover H_2_O from humidity.^18,19,21,28,39^ The H_2_O affinity of the unsolvated **BipyM** system strongly contrasts with that exhibited by the related compounds {Fe(pyrimidine)_2_[M(CN)_4_]} (M = Pt, Pd), which also display an available uncoordinated N atom in the axial pyrimidine ligands. In their monohydrated forms, the H_2_O molecule seems more labile, exhibiting full (M = Pt) and partial (M = Pd) spontaneous desorption. However, only the Pt derivative can recover half of the H_2_O molecule, while the Pd derivative does not show H_2_O adsorption in contact with air. This smaller affinity for H_2_O may be related to the much smaller interlayer distance and likely larger rigidity exhibited by the pyrimidine derivatives, which prevent the framework from hosting the H_2_O molecules.

The lack of structural modifications observed from the PXRD patterns upon soaking **PhPyM** in H_2_O (Figure S11) suggests a minimal adsorption of this guest. This observation is likely due to the hydrophobic nature of the intralayer cavities in these derivatives. In contrast, the PXRD patterns for these derivatives immersed in MeOH (Figure S11) denote remarkable changes in their structure, suggesting a greater tolerance toward MeOH adsorption, a fact that is likely due to the stronger affinity of MeOH to hydrophobic voids.^40^

### Nature of the Axial Ligand and Its Influence on the SCO

Although here we contribute to a detailed structural study and guest-dependent SCO experiments for compounds **PhPyPt** and **PhPyPd**, their magnetic behavior in the unsolvated form was already reported^35^ and successfully reproduced in this work. The formal replacement of 4-PhPy with 2,4-Bipy as the axial ligand in the 2D Hofmann-type {Fe(L)_2_[M(CN)_4_]} series provokes remarkable changes in the SCO behavior. As far as the isostructural unsolvated forms are concerned, the average *T*_1/2_ temperatures of the 2,4-Bipy derivatives are 38 K (M = Pt) and 44 K (M = Pd) larger than those of the 4-Phpy counterparts. Without excluding the possible influence of subtle structural effects, this marked difference should be mainly ascribed to the electronic effects induced by the peripheral 4-pyridyl or 4-phenyl groups on the coplanar coordinating pyridine ring. In this respect, although similar coordinating capabilities are expected for both ligands, the slightly larger electronegativity of 2,4-Bipy associated with the presence of the noncoordinating pyridine group, on the one hand, makes the N lone pair of the coordinating pyridine group less diffuse, thereby decreasing its σ-donor character but, on the other hand, simultaneously increases the π-acceptor character of 2,4-bipy, with the resulting effect being an increase of the ligand-field strength and, hence, the *T*_1/2_ values for the **BipyM** derivatives.

### Guest-Dependent SCO Behavior

Concerning the solvated forms, the changes observed in the SCO reflect the ability of the framework to include the guest molecules and the resulting host–guest interplay. For example, the **PhPyM** derivatives soaked in H_2_O exhibit SCO behaviors centered at temperatures only slightly smaller (5 K for M = Pd and 14 K for M = Pt) than the ones observed for the unsolvated counterparts (note that only *T*_1/2_^↓^ is reduced, whereas *T*_1/2_^↑^ is not modified). This suggests that the amount of adsorbed H_2_O is very small, prompting a slight stabilization of the HS via elastic frustration. In contrast, the **BipyM** derivatives soaked in H_2_O essentially recover the original SCO of the as-synthesized samples. It is worth mentioning that the *T*_1/2_ values of the **BipyM·H**_**2**_**O** solvates are in the range of 50–60 K higher than those of their unsolvated counterparts. This important stabilization of the LS state shifting *T*_1/2_ near room temperature must be facilitated by the highly regular orthorhombic *Cmmm* structure adopted by **BipyM·H**_**2**_**O**, which favors the lack of elastic frustration. The increase of *T*_1/2_ with the adsorption of H_2_O is relatively common in 3D Hofmann-type compounds and has been associated with structural changes produced upon the hydration process and/or the host–guest interactions (i.e., hydrogen bonding) between H_2_O and the ligands surrounding the Fe^II^ ion.^15^ However, a *T*_1/2_ increase mediated by H_2_O adsorption is rare in 2D Hofmann structures. This is the case of compound {Fe(thtrz)_2_[Pd(CN)_4_]}, for which a difference of 40 K between the empty and monohydrated derivatives was also explained by host–guest interactions influencing the Fe^II^ environment.^19^ Conversely, the majority of the reported 2D-layered Hofmann structures present an increase of *T*_1/2_ upon dehydration, which has been mainly justified by a reduction of the elastic frustration. In the present case, inclusion of the H_2_O molecule not only is not a source of steric hindrance but also is responsible for the *T*_1/2_ upshift until attaining the room temperature range. Importantly, whatever the degree of solvation, examples of 2D Hofmann frameworks presenting hysteretic SCO properties centered around room temperature are still scarce^18,24^ and are needed for future applications.

The progressive and controlled desorption of H_2_O in **BipyM·H**_**2**_**O** to afford **BipyM** is accompanied by a reduction in *T*_1/2_ and the occurrence of a crystallographic transformation from the orthorhombic *Cmmm* to the monoclinic *I*2/*m* space group. The latter defines a less regular network, which is reflected in an important increase of corrugation in the bimetallic layers. Interestingly, this decrease in *T*_c_ does not seem to be homogeneous with the sample showing intermediate dehydrated phases, whose SCO behaviors suggest the presence of mixtures of pure hydrated and dehydrated species. However, interestingly, the evolution of this dehydration process gives rise to an intermediate phase that displays a huge square-shaped hysteresis loop 84 K, which clearly suggests the presence of a homogeneous genuine phase instead the overlap of two distinct phases. The presence of such an intermediate state was detected through sophisticated single-crystal X-ray diffraction analysis in the mononuclear system [Fe(bpp)(H_2_L)](ClO_4_)_2_·1.5C_3_H_6_O [bpp = 2,6-bis(pyrazol-3-yl)pyridine; H_2_L = 2,6-bis[5-(2-methoxyphenyl)pyrazol-3-yl]pyridine; C_3_H_6_O = acetone].^41^

The introduction of MeOH in both the **PhPyM** and **BipyM** frameworks induces deactivation of the SCO. Indeed, the magnetic measurements of these compounds soaked in liquid MeOH yield paramagnetic curves. This result is consistent with the structures of **PhPyM·MeOH·0.5H**_**2**_**O** and **BipyPt·MeOH·H**_**2**_**O**, where the Fe^II^ ions are HS at all temperatures. As mentioned above, the adsorption of MeOH implicates important structural changes in the host framework, i.e., the adjacent axial ligand 2,4-Bipy (or 4-PhPy) of a layer tilt in opposite directions, opening channels along which the MeOH is accommodated. These structural changes generate short intermolecular contacts featuring steric repulsions, i.e., those established between the methylene H atoms of MeOH and the aromatic H atoms of the coordinating pyridine. Likely, this steric factor is responsible for the elastic frustration that involves stabilization of the HS state. Along this line, the small fraction of SCO-active Fe^II^ ions observed for crystals of **FePhPy·*x*MeOH·*y*H**_**2**_**O** (Figure 1b) soaked in the mother liquor is likely due to the existence of MeOH-free phases containing H_2_O or exhibiting complete absence of solvent. Indeed, the presence of this SCO-active phase was detected by PXRD (Figure S1).

## Conclusions

In summary, guest-dependent structural and SCO properties of the isomorphous 2D Hofmann CPs {Fe(4-PhPy)_2_[M(CN)_4_]} (**PhPyM**) and {Fe(2,4-Bipy)_2_[M(CN)_4_]} (**BipyM**) (M = Pd, Pt) were characterized and compared. Our results indicate that, although the sole difference between both families of compounds stems from the presence/absence of a noncoordinated N heteroatom within the interlayer space, this subtle difference in the chemical composition deeply impacts not only the adsorption capability of the clathrate but also the degree to which the SCO is modulated upon guest uptake. Thus, whereas the adsorption of H_2_O in **BipyM** is rapid and spontaneous at ambient conditions, it occurs to a very low extent for **PhPyM**, even when soaked in H_2_O. The driving force of the higher H_2_O affinity of the former is necessarily ascribed to the hydrogen bonds established between H_2_O and the noncoordinated N heteroatom. These interactions induce the opening of interlayer channels along which the H_2_O molecules are accommodated, leading to regular and homogeneous layered structures. The uptake of H_2_O in **BipyM** provokes a remarkable increase of the SCO temperatures (by 50–60 K), shifting the wide hysteresis loops at around room temperature. This SCO shift has been correlated with a combination of structural and electronic factors. In contrast, the small amount of H_2_O adsorbed by **PhPyM** marginally influences the SCO temperature, likely due to subtle elastic frustration effects. Besides, although MeOH molecules are efficiently absorbed in solution by both series of compounds leading to distorted layered networks, steric repulsions between the MeOH molecules and host network are behind its rapid desorption under an ambient atmosphere. This steric factor also provides elastic frustration to the system because the SCO properties of both families of compounds are deactivated when soaked in MeOH. This comparative study sheds light on how fine-tuning the host framework greatly modifies the coupling of host–guest and SCO properties and contributes to paving the way toward the application of these materials as chemical sensors.

## Supporting Information Available

Crystallographic details, additional structural views and PXRD patterns, Rietveld refinement plots, TGA data, and additional magnetic measurements (PDF)

## Accession Codes

CCDC 2090594–2090601 contain the supplementary crystallographic data for this paper. These data can be obtained free of charge via www.ccdc.cam.ac.uk/data_request/cif, or by emailing data_request@ccdc.cam.ac.uk, or by contacting The Cambridge Crystallographic Data Centre, 12 Union Road, Cambridge CB2 1EZ, UK; fax: +44 1223 336033.
