# Supplementary material for: Enhanced Interplay between Host–Guest and Spin-Crossover Properties through the Introduction of an N Heteroatom in 2D Hofmann Clathrates
Source: Inorg Chem. 2021 Aug 4;60(16):11866–77. doi: 10.1021/acs.inorgchem.1c01925 (PMC9490864; doi:10.1021/acs.inorgchem.1c01925)

Supporting information

Enhanced interplay between host-guest and Spin Crossover properties through introduction of a N-heteroatom in 2D Hofmann clathrates

Manuel Meneses-Sánchez,[a] Rubén Turo-Cortés,[a] Carlos Bartual-Murgui,*[a] Iván da Silva,[b] M. Carmen Muñoz,[c] J. Antonio Real*[a]

[a] Instituto de Ciencia Molecular/Departamento de Química Inorgánica, Universidad de Valencia, Catedratico Beltrán Martínez 2, E-46980 Paterna, València, Spain. Email: carlos.bartual@uv.es; jose.a.real@uv.es.

[b] ISIS Neutron Facility, STFC Rutherford Appleton Laboratory, Chilton, Oxfordshire OX11 0QX, UK

[c] Departamento de Física Aplicada, Universitat Politècnica de València, Camino de Vera S/N 46022 Valencia, Spain.

Figure S1. Experimental PXRD spectra at room temperature for **PhPyPt·xMeOH·yH_2O**, **PhPyPd·xMeOH·yH_2O** and PXRD simulated for **PhPyM·MeOH·0.5H_2O.**

Figure S2. Thermogravimeric analyses for **PhPyM·xMeOH·yH_2O** (M = Pt, Pd).

Figure S3. PXRD spectra at room temperature for **BipyM·H_2O** (M = Pt, Pd).

Figure S4. Thermogravimeric analyses of **BipyM·H_2O** (M = Pt, Pd).

Figure S5. Time dependent TGA for **BipyM** (M = Pt, Pd).

Figure S6. Experimental PXRD spectra at room temperature for **PhpyM** and **BipyM** (M = Pt, Pd).

Figure S7-S8. Magnetic properties of **PhPyM·xMeOH·yH_2O and PhpyM** (M = Pt, Pd) in different conditions.

Figure S9-10. Magnetic properties of **BipyM·xH_2O** (M = Pt, Pd) in different conditions.

Figure S11. Experimental and simulated PXRD spectra of **PhpyM** and **PhPyM·MeOH·0.5H_2O** (M = Pt, Pd), respectively.

Figure S12. Experimental and simulated PXRD spectra of **BipyM** and **BipyPt·H_2O·MeOH** (M = Pt, Pd), respectively.

Figure S13-14. Magnetic properties of **PhPyM** and **BipyM** (M = Pt, Pd) soaked in water or methanol.

Figure S15. Thermogravimetric analyses of **PhPyM**, **BipyM** (M = Pt, Pd) upon removing from H_2O and MeOH, respectively, and exposed to air.

Figure S16. DSC measurements for compound **BipyPt·H_2O**.

Figure S17. Final Rietveld refinement plots for compounds (a) **PhPyM** and (b) **BipyM.**

Figure S18. Views of the structures of **PhPyM·MeOH·0.5H_2O** and **BipyM·H_2O** along the (100) and (001) directions, respectively.

Table S1. Crystal data for **PhPyM·MeOH·0.5H_2O** at 120 K (M = Pt, Pd).

Table S2. Crystal data for **BipyPt·H_2O** at 120 K and 283 K, **BipyPt·H_2O·MeOH** at 120 K and **BipyPd·H_2O** at 120 K.

Table S3. Crystallographic data and Rietveld refinement summary for compounds **PhPyPt** and **BipyPt**.

Table S4. Selected bond lengths and angles for **PhPyM·MeOH·0.5H_2O** (M = Pt, Pd) at 120K

Table S5. Selected bond lengths [Å] and angles [°] **BipyPt·H_2O** at 120 K and 283 K, **BipyPt·H_2O·MeOH** at 120 K and **BipyPd·H_2O** at 120 K.

Table S6. π–π interaction and H-bonding distances found for the different compounds.

Figure S1. Powder X-ray diffraction spectra at room temperature of a) **PhPyPt·xMeOH·yH2O** and b) **PhPyPd·xMeOH·yH2O** measured in their mother liquor (black) and after exposed to air and air-dried for several minutes (red) and (c and d) their corresponding enlarged 6.5-8° windows. Simulated spectra of the solvates **PhPyM·MeOH·0.5H2O** are also shown as reference.

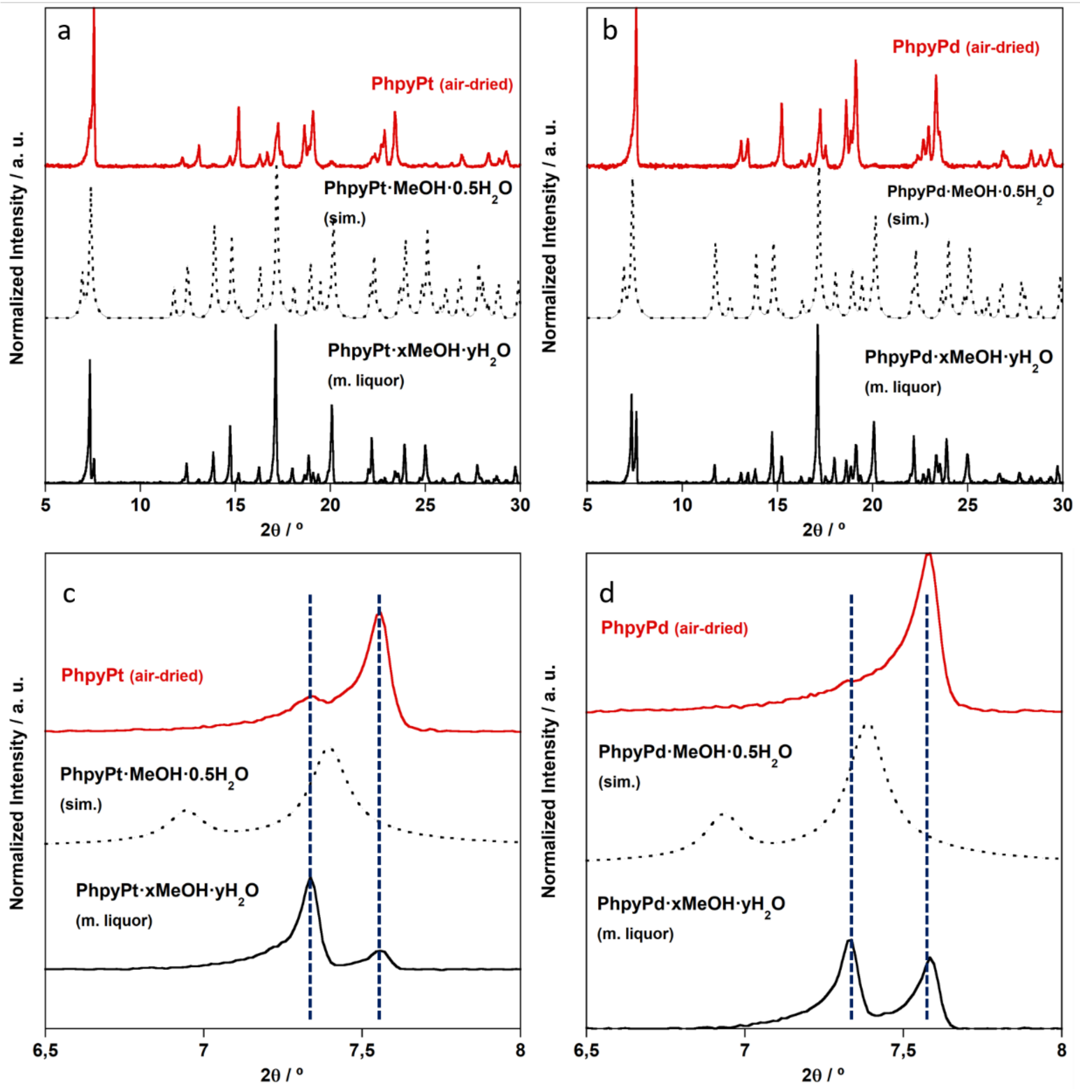

Figure S2. Thermogravimetric analyses of **PhPyM·xMeOH·yH2O** (a) M = Pt and b) M = Pd) recorded upon removing the crystals from mother liquor and air dried at room temperature (blue) and several days after heating the samples at 400 K for 1 h and re-exposed to air (red).

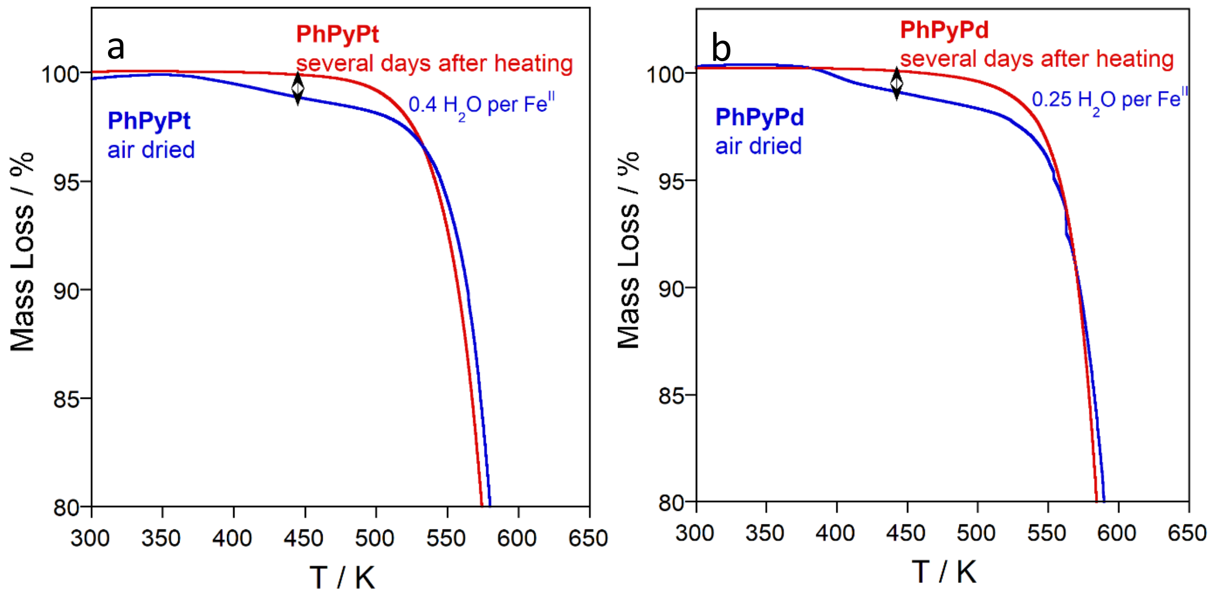

Figure S3. Powder X-ray diffraction spectra at room temperature of a) **BipyPt·H2O** and b) **BipyPd·H2O** measured in their mother liquor (black), after exposed to air and air-dried at room temperature for several minutes (blue) and once heated at 400 K for one hour (red). Simulated spectra of compounds **BipyM·H2O** are also shown as reference.

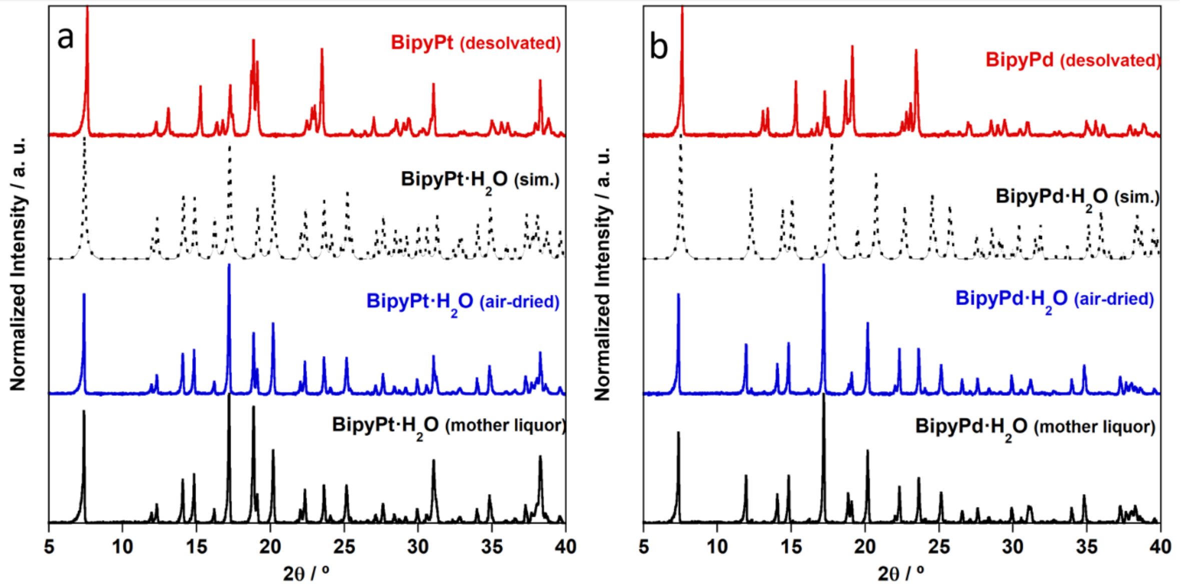

Figure S4. Thermogravimetric analyses of **BipyM·H_2O** (M = Pt (red) and Pd (blue)) recorded upon removing the crystals from mother liquor and air dried at room temperature.

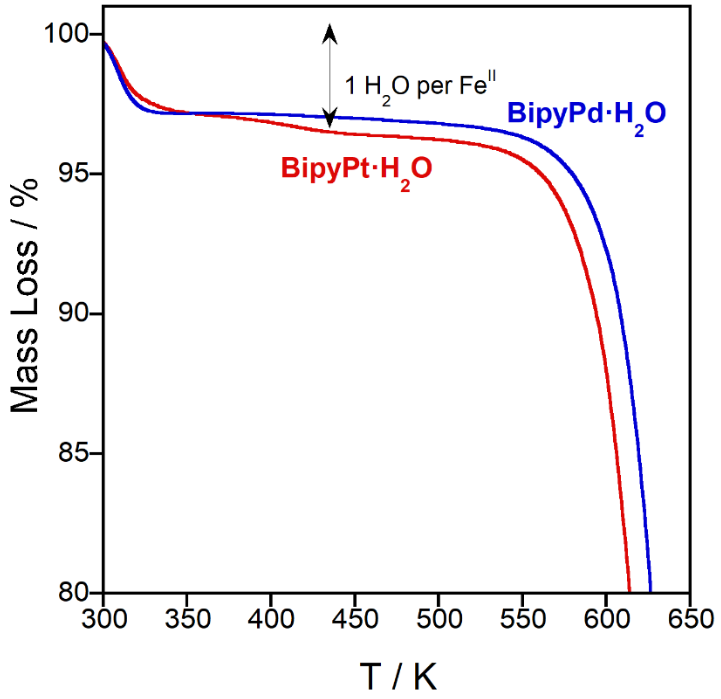

Figure S5. Time dependent TGA measurements registered upon desorption and reabsorption of water for a) **BipyPt** and b) **BipyPd**.

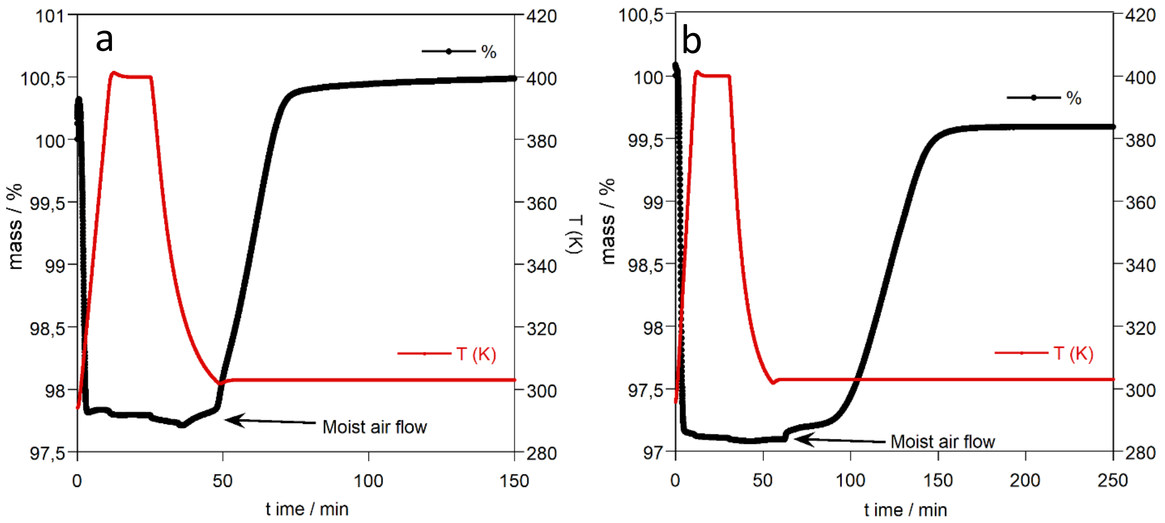

Figure S6. Powder X-ray diffraction spectra at room temperature of the desolvated networks based on the 2,4-Bipy (black) or 4-PhPy (blue) ligands.

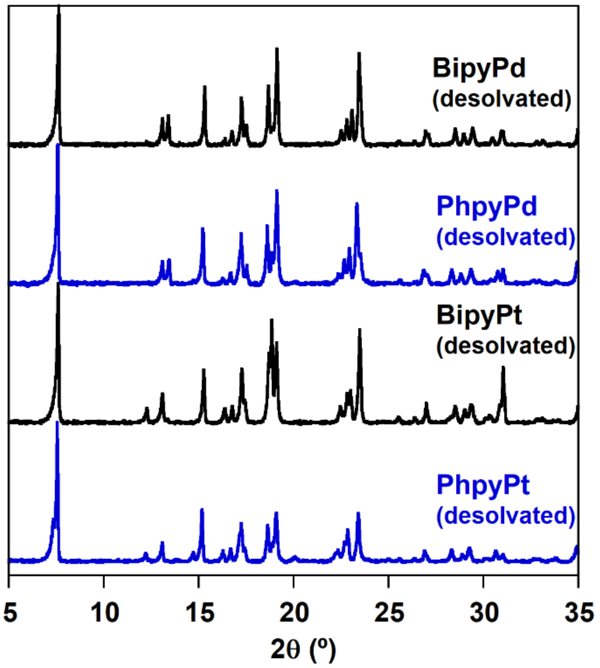

Figure S7. Thermal dependence of the $\chi_M T$ value for compounds **PhPyM·xMeOH·yH2O** (M = Pt (a) or Pd (b)) measured in the mother liquor (black), once extracted from the mother liquor and air dried (blue) and after a treatment at 400 K for 1 hour (red).

Fi

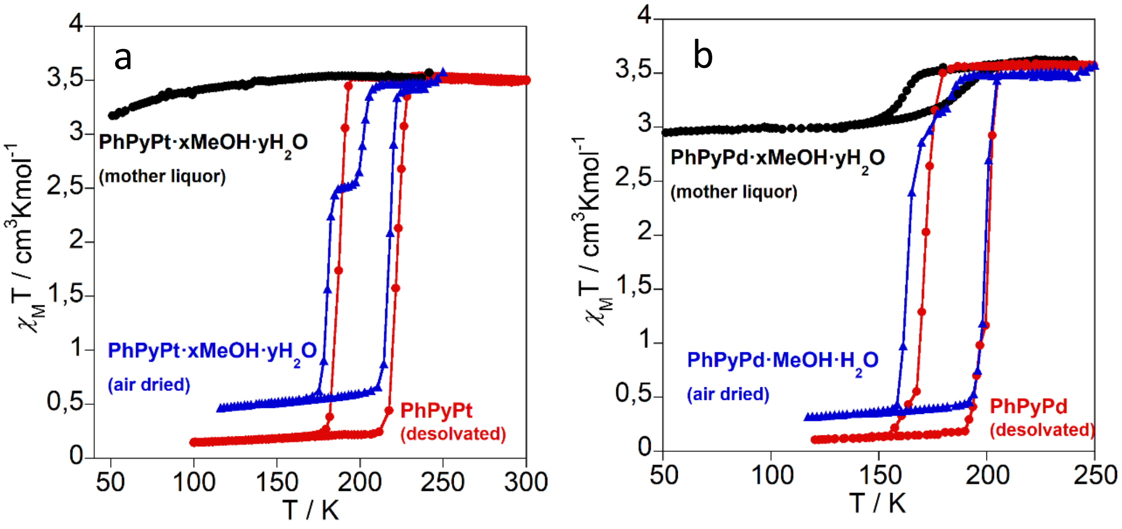

Figure S8. Thermal dependence of the $\chi_M T$ value for compounds a) **PhPyPt** and b) **PhPyPd** just after dehydrating at 400 K for 1 h (red) and after exposing the sample to air for several days (blue).

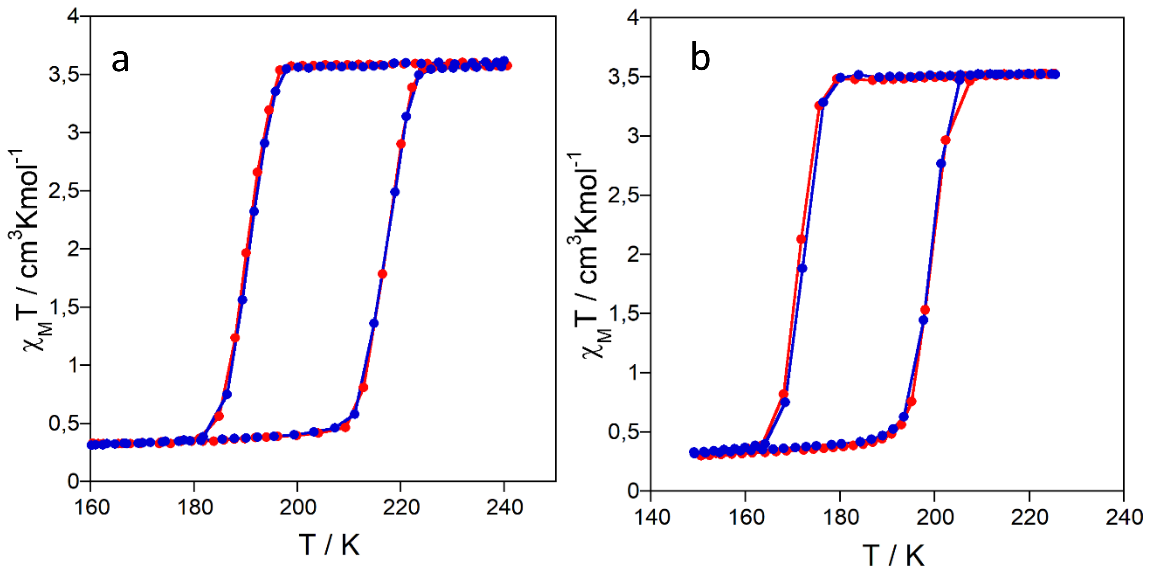

Figure S9. Thermal dependence of the $\chi_M T$ value for compounds a) **BipyPt·H$_2$O** and b) **BipyPd·H$_2$O** after removing the crystals from the mother liquor and air drying at ambient conditions.

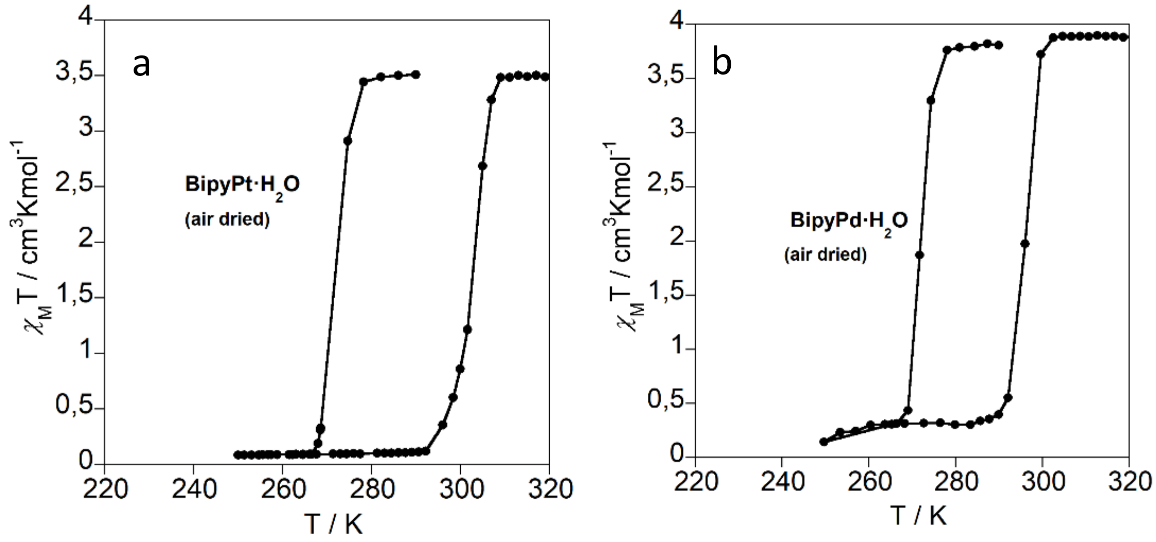

Figure S10. Thermal dependence of the $\chi_M T$ value for dehydrated compounds a) **BipyPt** and b) **BipyPd** after ca. one hour exposed to air. The resulting SCO curves are similar to those of **BipyPt·H2O** and **BipyPd·H2O** indicating that the dehydrated phase recovers water in these conditions.

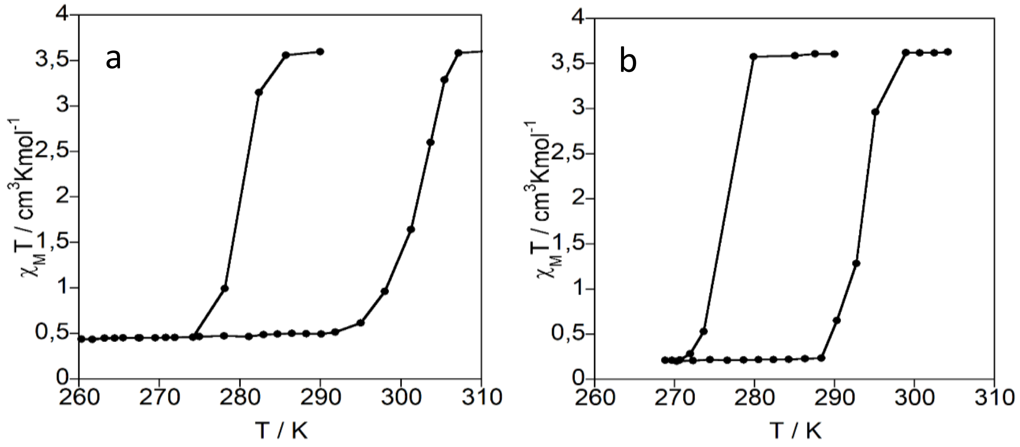

Figure S11. Powder X-ray diffraction patterns at room temperature of compounds a) **PhPyPt** and b) **PhPyPd** after dehydrating at 400 k for 1h (red), immersed in water (blue) or methanol (black). The simulated spectra of solvates **PhPyPt·MeOH·0.5H2O** and **PhPyPd·MeOH·0.5H2O** are also displayed for comparison.

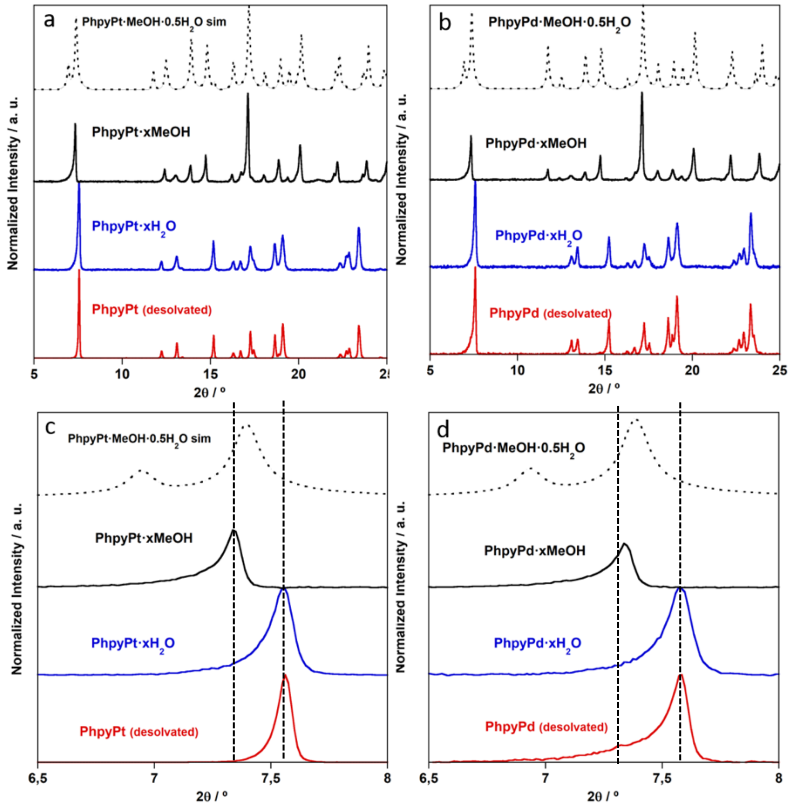

Figure S12. Powder X-ray diffraction patterns at room temperature of compounds a) **BipyPt** and b) **BipyPd** after dehydrating at 400 k for 1h (red), immersed in water (blue) or methanol (black). The simulated spectra of the solvate **BipyPt·H2O·MeOH** is also displayed for comparison.

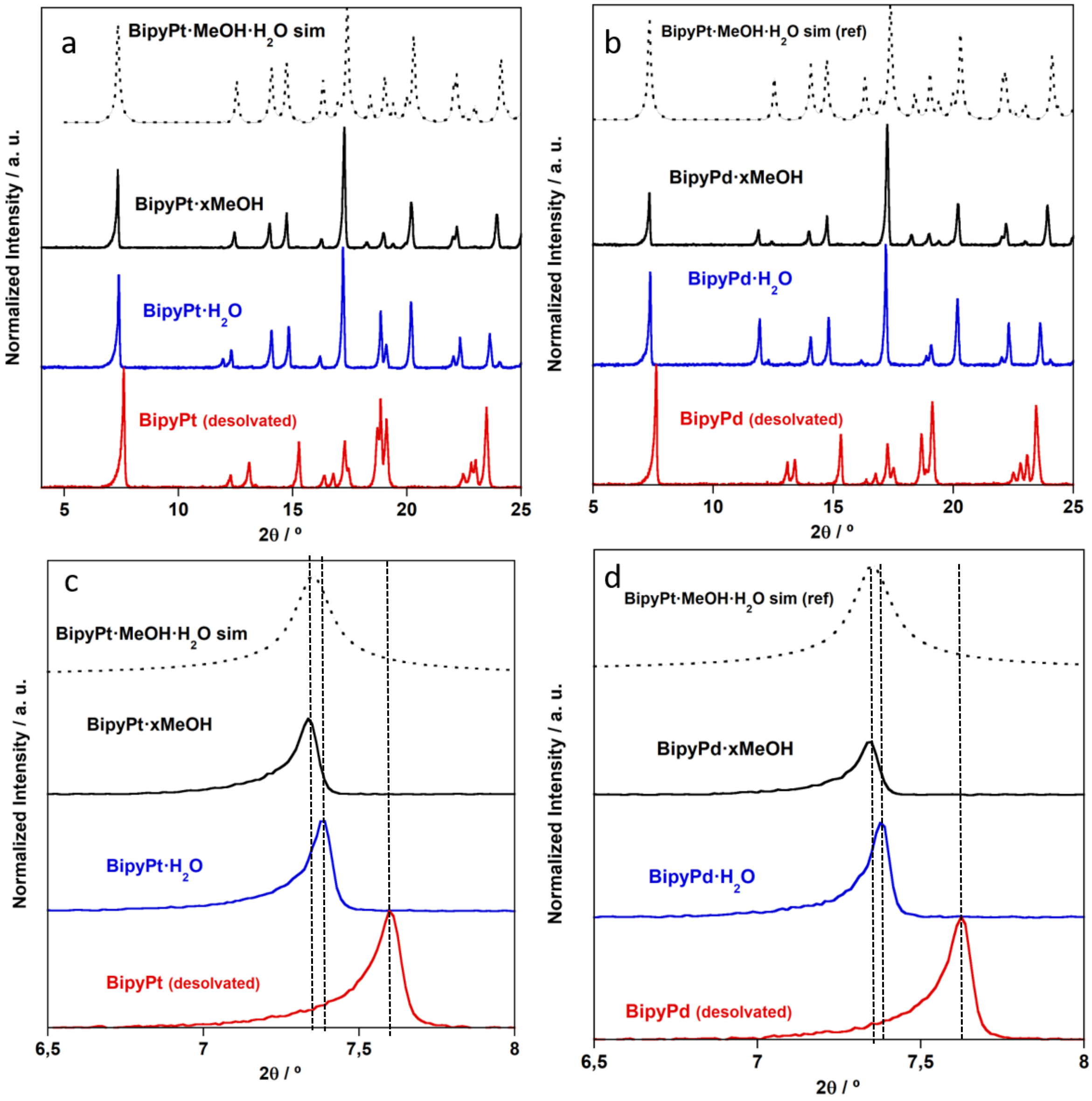

Figure S13. SCO properties of a) **PhPyPd** and b) **BipyPd** before (black curves) and after absorbing water (blue curves) and methanol (red curves). SCO red and blue curves were registered with the samples soaked in the corresponding solvent.

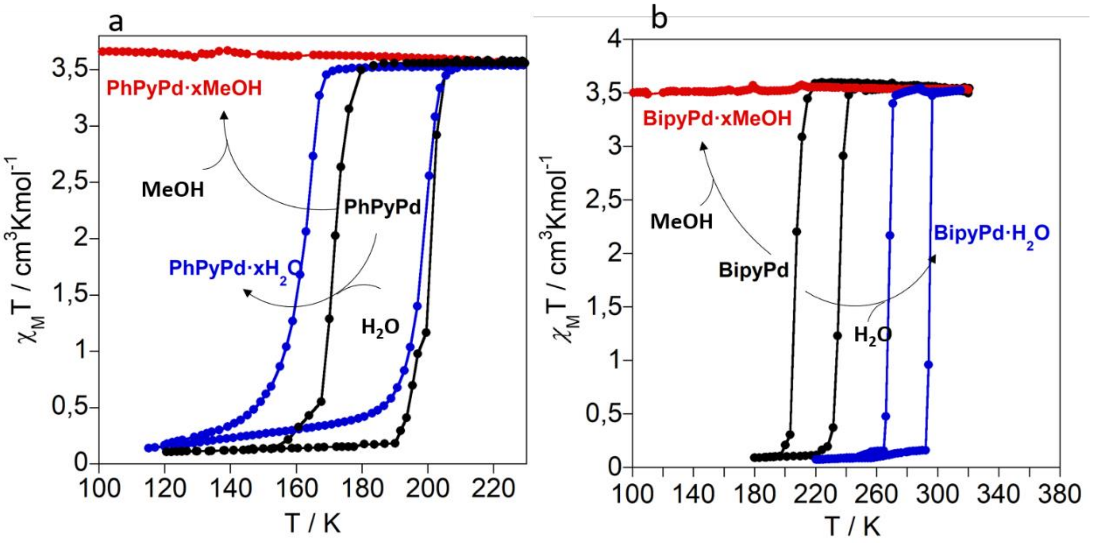

Figure S14. SCO properties of **PhPyPt** (a and b) and **PhPyPd** (d and e) inmersed in H_2O and MeOH (black curves), respectively, and after removing the crystals from the corresponding solvent and exposed to air (blue curves). SCO of c) **BipyPt** and f) **BipyPd** inmersed in MeOH (black curves) and after removing from methanol and exposed to air (blue curves).

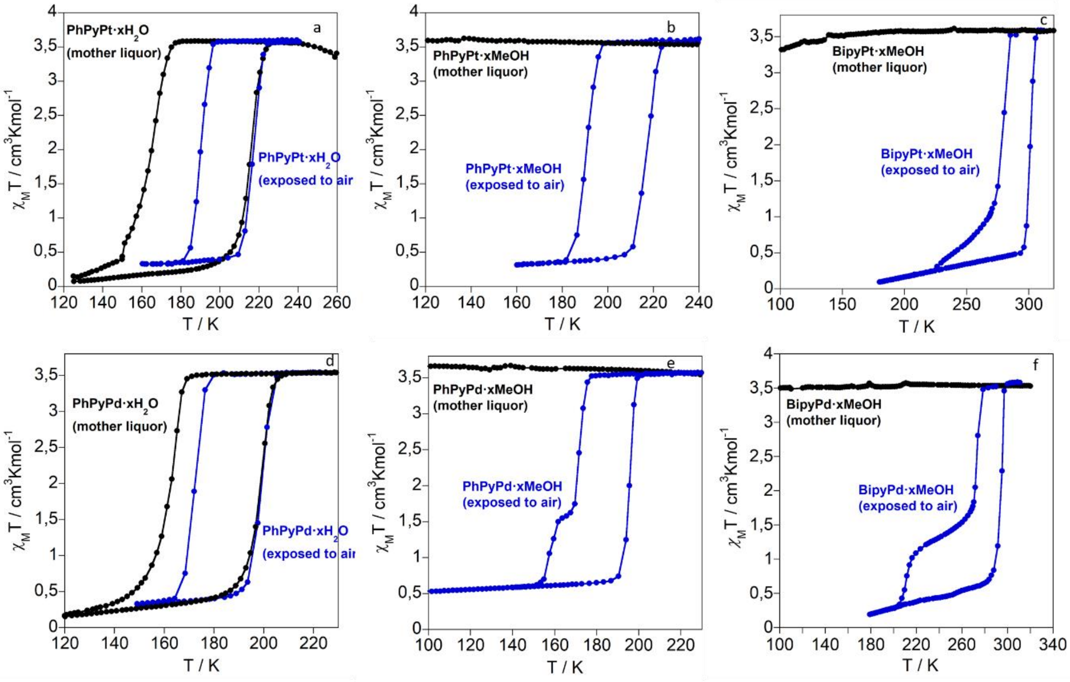

Figure S15. Thermogravimetric analyses of **PhPyPt** (a and b), **BipyPt** (c and d) **PhPyPd** (e and f), and **BipyPd** (g and h) upon removing from H_2O and MeOH, respectively, and exposed to air. Note that, in view of the magnetic properties, the mass loss registered in curves d and h is mostly associated to water molecules that has been interchanged by those of methanol at ambient conditions.

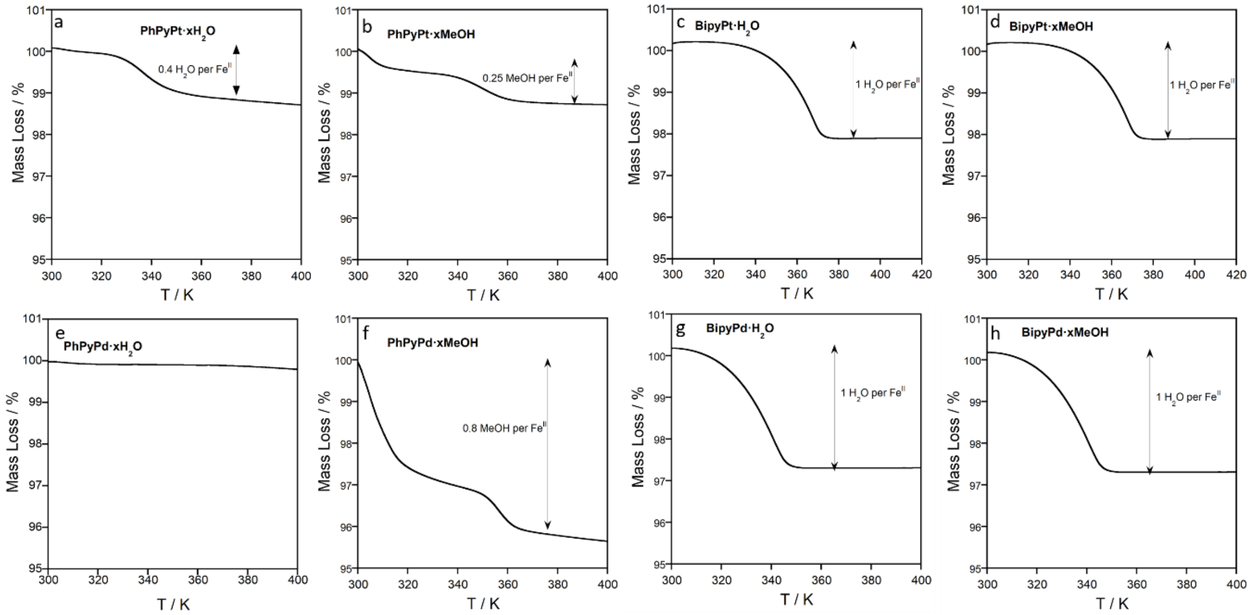

Figure S16. DSC measurements for compound **BipyPt·H2O** registered after heating inside the calorimeter device at 305 K for a few minutes. Blue and red curves represent the cooling and heating modes, respectively. Magnetic measurements of a **BipyPt·H2O** sample subjected to a similar heating treatment are displayed for comparison.

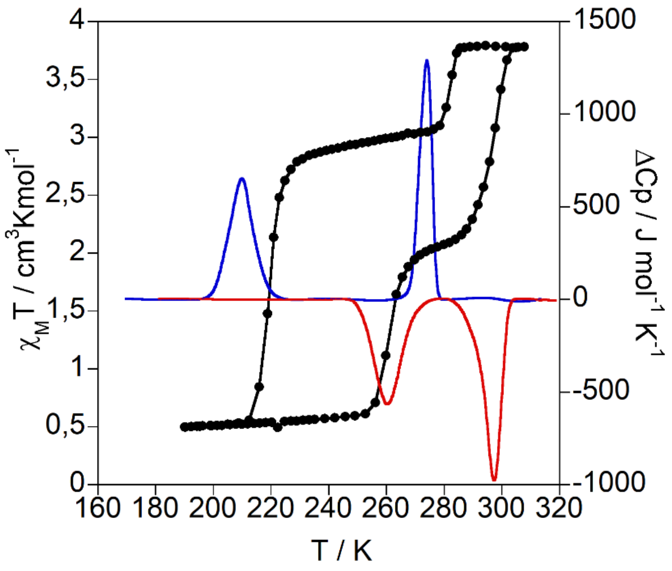

Table S1. Crystal data for **PhPyM·MeOH·0.5H$_2$O** at 120 K (M = Pt, Pd).

	**PhPyPt·MeOH·0.5H$_2$O**	**PhPyPd·MeOH·0.5H$_2$O**
Empirical formula	$C_{27}H_{18}N_6O_{1.5}PtFe$	$C_{27}H_{18}N_6O_{1.5}PdFe$
*M*r	701.41	612.72
Crystal system	orthorhombic	orthorhombic
Space group	*Imma*	*Imma*
a (Å)	15.0241(4)	15.0473(3)
b (Å)	7.4189(2)	7.4095(2)
c (Å)	23.8844(6)	23.9166(5)
V (Å^3)	2662.21(12)	2666.53(11)
Z	4	4
D_c (mg cm^{-3})	1.750	1.526
F(000)	1352	1224
μ (Mo-K$_\alpha$) (mm^{-1})	5.826	1.251
Crystal size (mm)	0.01x0.08x0.08	0.01x0.12x0.12
No. of total reflections	1815	1699
No. of reflections [I>2$\sigma(I)$]	1808	1620
R [I>2$\sigma(I)$]	0.0175	0.0353
wR [I>2$\sigma(I)$]	0.0483	0.0977
S	1.171	1.126

Table S2. Crystal data for **BipyPt·H$_2$O** at 120 K and 283 K, **BipyPt·H$_2$O·MeOH** at 120 K and **BipyPd·H$_2$O** at 120 K.

	**BipyPt·H$_2$O_120 K**	**BipyPt·H$_2$O_283 K**	**BipyPt·H$_2$O·MeOH_120K**	**BipyPd·H$_2$O_120K**
Empirical formula	$C_{24}H_{18}N_8OPtFe$	$C_{24}H_{16}N_8OPtFe$	$C_{25}H_{20}N_8O_2PtFe$	$C_{24}H_{18}N_8OPdFe$
*M*r	685.40	683.39	715.43	596.71
Crystal system	orthorhombic	orthorhombic	orthorhombic	orthorhombic
Space group	*Cmmm*	*Cmmm*	*Pnma*	*Cmmm*
a (Å)	7.2122(3)	7.5176(8)	14.754(4)	7.2266(2)
b (Å)	23.3642(9)	23.803(2)	7.370(2)	23.4174(5)
c (Å)	7.1140(3)	7.3695(7)	24.028(8)	7.1253(2)
V (Å^3)	1198.76(8)	1318.7(2)	2612.8(13)	1205.80(5)
Z	2	2	4	2
D_c (mg cm^{-3})	1.899	1.721	1.819	1.643
F(000)	660	656	1384	596
μ (Mo-K$_\alpha$) (mm^{-1})	6.468	5.879	5.942	1.381
Crystal size (mm)	0.02x0.12x0.12	0.02x0.12x0.12	0.02x0.10x0.10	0.04x0.12x0.12
No. of total reflections	716	789	2999	820
No. of reflections [I>2$\sigma(I)$]	710	675	2466	740
R [I>2$\sigma(I)$]	0.0337	0.0675	0.0797	0.0253
wR [I>2$\sigma(I)$]	0.0600	0.1343	0.2041	0.0526
S	1.091	1.103	1.235	1.080

Table S3. Crystallographic data and Rietveld refinement summary for compounds **PhPyPt** and **BipyPt**.

Compound	**BipyPd**	**BipyPt**
Empirical formula	$(C_{11}H_9N)_2$ $(CN)_4$ Fe Pt	$(C_{10}H_8N_2)_2$ $(CN)_4$ Fe Pt
Formula weight (g/mol)	665.39	667.38
Temperature, K	298	298
Crystal system	Monoclinic	Monoclinic
Space group	*I* 2/*m*	*I* 2/*m*
a, Å	6.96068(12)	6.96916(12)
b, Å	7.58951(10)	7.56020(11)
c, Å	23.3210(4)	23.1696(4)
b, °	92.4728(13)	92.2282(14)
Volume, Å^3	1230.86(3)	1219.84(4)
Z	2	2
Radiation	Cu K$\alpha_{1,2}$	Cu K$\alpha_{1,2}$
2θ range, °	5 – 90	5 – 90
R_p, %	2.26	2.45
R_{wp}, %	3.18	3.33
R_{exp}, %	2.26	2.45
GoF	1.40	1.36
Fe-N(1)	2.224(7)	2.222(7)
Fe-N(4)	2.141(10)	2.140(12)
Pt-C(12)	1.983(14)	1.983(18)
N(1)-Fe-N(4)	90.9(4)	91.0(5)
Cl(12)-Pt-Cl(12)	180.00	180.00

Table S4. Selected bond lengths [Å] and angles [°] for **PhPyM·MeOH·0.5H$_2$O** (M = Pt, Pd) at 120K.

	**PhPyPt**	**PhPyPd**
Fe-N(1)	2.157(2)	2.159(3)
Fe-N(2)	2.223(2)	2.225(3)
M-C(1)	1.989(3)	1.993(3)
N(1)-Fe-N(2)	90.10(12)	90.21(14)
C(1)-M-C(1)	178.90(12)	178.7(2)

Table S5. Selected bond lengths [Å] and angles [°] **BipyPt·H$_2$O** at 120 K and 283 K, **BipyPt·H$_2$O·MeOH** at 120 K and **BipyPd·H$_2$O** at 120 K.

	**bipyPt·H$_2$O_120K**	**bipyPt·H$_2$O_283K**	**bipyPt·H$_2$O·MeOH_120K**	**bipyPd·H$_2$O_120K**
Fe-N(1)	1.932(5)	2.133(13)	2.122(14)	1.937(2)
Fe-N(2)	1.993(7)	2.225(19)	2.16 (2)	2.000(3)
Fe-N(3)			2.163(14)	
Fe-N(4)			2.24(2)	
M-C(1)	1.989(6)	1.993(15)	1.99(2)	1.991(3)
M-C(2)			2.00(2)	
N(1)-Fe-N(2)	90.0	90.0	92.6(5)	90.0
N(1)-Fe-N(3)			176.9(6)	
N(1)-Fe-N(4)			90.0(5)	
N(2)-Fe-N(3)			90.4(5)	
N(2)-Fe-N(4)			176.4(7)	
N(3)-Fe-N(4			87.1(6)	
C(1)-M-C(1)	180.0	180.0		180.0
C(1)-M-C(2)			178.8(6)	

Table S6. π–π interaction and H-bonding distances [Å] found for the different compounds.

	π–π		H-bonding	
	**LS**	**HS**	**HO-H···N(pyridine)**	**MeO-H···N(pyridine)**
**PhPyPt·MeOH·0.5H_2O**	-	3.720	-	-
**PhPyPd·MeOH·0.5H_2O**	-	3.740	-	-
**BipyPt·H_2O**	3.665	3.786	2.953(LS)-3.202(HS)	-
**BipyPd·H_2O**	3.671	-	3.000(LS)	-
**BipyPt·H_2O·MeOH**	-	3.722	3.331	2.835
**BipyPt**	-	3.988	-	-
**PhPyPt**	-	4.003	-	-

Figure S17. Final Rietveld refinement plots for compounds (a) **PhPyM** and (b) **BipyM**, showing the experimental (red circles), calculated (black line) and difference profiles (grey line); blue marks indicate reflection positions.

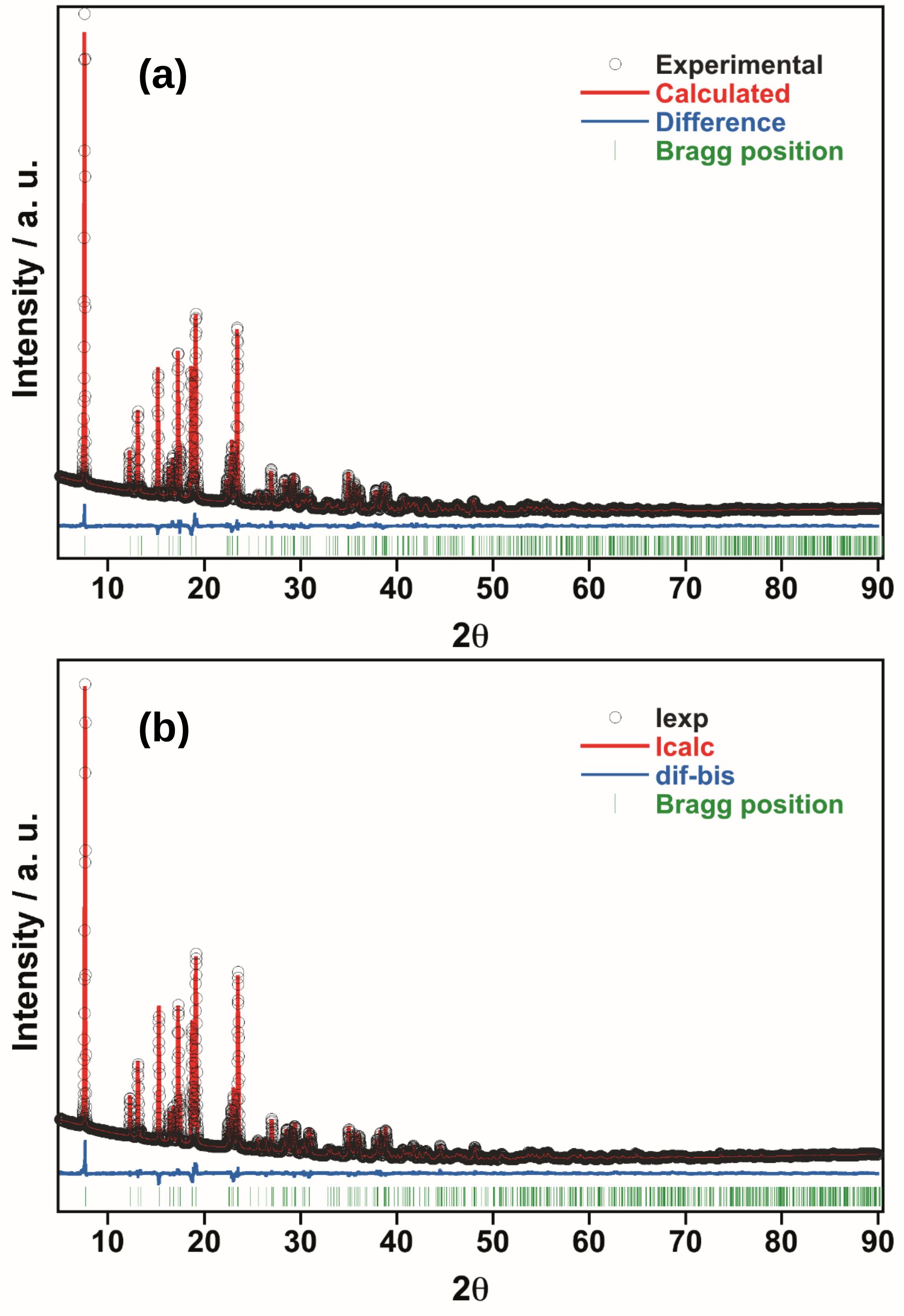

Figure S18. Views of the structures of a) **PhPyM·MeOH·0.5H$_2$O** and **BipyM·H$_2$O** along the (100) and (001) directions, respectively, showing the interdigitation of four consecutive bimetallic layers.

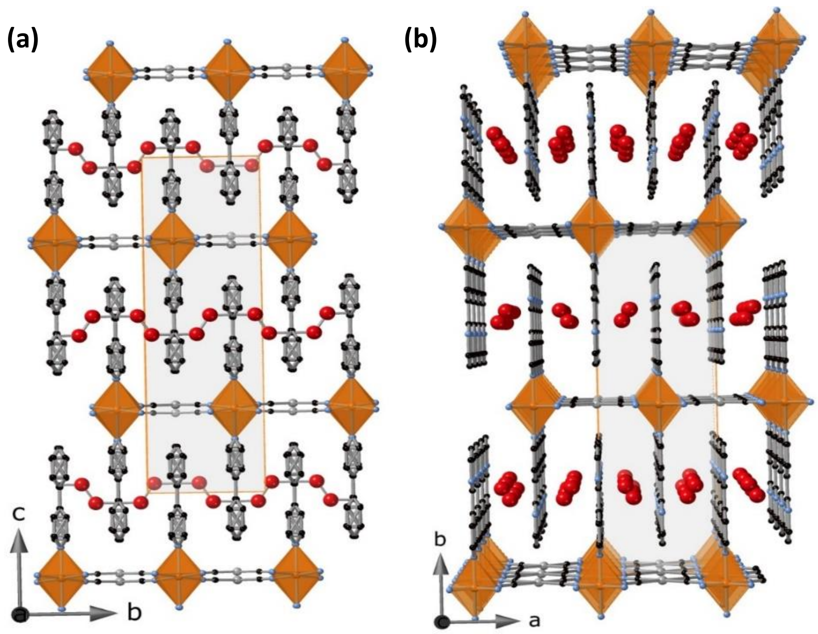

Supplement: Supplementary file 1 — ic1c01925_si_002.pdf [file ic1c01925_si_002.pdf]
